# Aggregation Behavior of Structurally Similar Therapeutic Peptides Investigated by ^1^H NMR and All-Atom Molecular Dynamics Simulations

**DOI:** 10.1021/acs.molpharmaceut.1c00883

**Published:** 2022-02-01

**Authors:** Johanna Hjalte, Shakhawath Hossain, Andreas Hugerth, Helen Sjögren, Marie Wahlgren, Per Larsson, Dan Lundberg

**Affiliations:** † Food Technology, Engineering and Nutrition, Lund University, Box 124, 221 00 Lund, Sweden; ‡ Department of Pharmacy, Drug Delivery, Uppsala University, Box 580, 751 23 Uppsala, Sweden; § Ferring Pharmaceuticals A/S, Amager Strandvej 405, 2770 Kastrup, Denmark; ∥ CR Competence AB, Center for Chemistry and Chemical Engineering, Box 124, 221 00 Lund, Sweden

**Keywords:** therapeutic peptides, aggregation, AA-MD simulations, ^1^H NMR spectroscopy, evaluation of developability

## Abstract

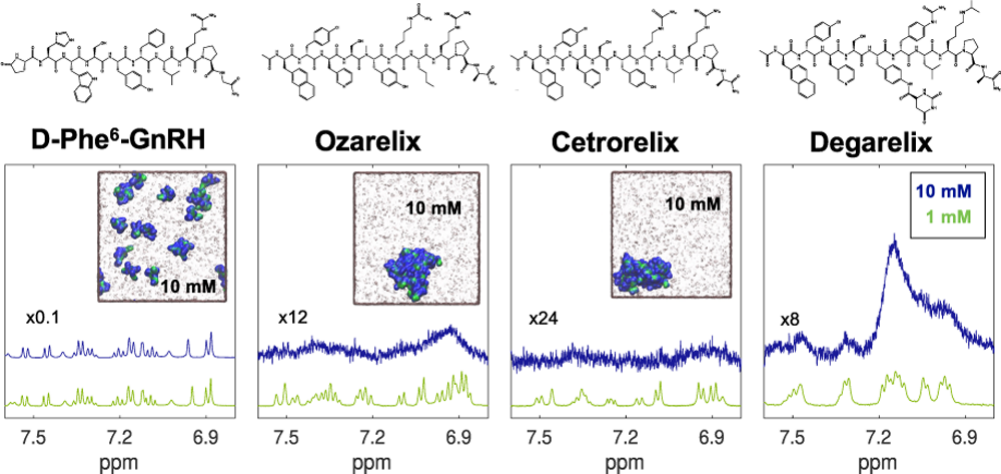

Understanding of peptide aggregation propensity is an important aspect in pharmaceutical development of peptide drugs. In this work, methodologies based on all-atom molecular dynamics (AA-MD) simulations and ^1^H NMR (in neat H_2_O) were evaluated as tools for identification and investigation of peptide aggregation. A series of structurally similar, pharmaceutically relevant peptides with known differences in aggregation behavior (D-Phe^6^-GnRH, ozarelix, cetrorelix, and degarelix) were investigated. The ^1^H NMR methodology was used to systematically investigate variations in aggregation with peptide concentration and time. Results show that ^1^H NMR can be used to detect the presence of coexisting classes of aggregates and the inclusion or exclusion of counterions in peptide aggregates. Interestingly, results suggest that the acetate counterions are included in aggregates of ozarelix and cetrorelix but not in aggregates of degarelix. The peptides investigated in AA-MD simulations (D-Phe^6^-GnRH, ozarelix, and cetrorelix) showed the same rank order of aggregation propensity as in the NMR experiments. The AA-MD simulations also provided molecular-level insights into aggregation dynamics, aggregation pathways, and the influence of different structural elements on peptide aggregation propensity and intermolecular interactions within the aggregates. Taken together, the findings from this study illustrate that ^1^H NMR and AA-MD simulations can be useful, complementary tools in early evaluation of aggregation propensity and formulation development for peptide drugs.

## 1 Introduction

The aggregation behavior of therapeutic peptides influences several critical aspects of pharmaceutical development, such as the dosage forms possible to develop, ease of manufacturing, formulation stability, and patient safety and convenience.^1−5^ Self-assembly is often an unwanted effect but can also be utilized to alter the pharmacokinetics of peptide and protein drugs^6−8^ and can improve the chemical and physical stability of a drug.^9^ When peptides self-assemble, they can, similarly to proteins, form various types of aggregates. The two most commonly discussed aggregate types for therapeutic peptides are (1) amyloid-like fibrils, where the peptides are folded into stacked β-sheets, and (2) amorphous aggregates, that is, disordered structures.^5,8,10,11^ Amyloid-like fibrils are known to be preceded by smaller aggregates, for example, oligomers or fibril fragments (protofibrils or filaments),^5^ and some peptides can form stable, well-defined oligomers.^12^ It is likely that transient oligomers, which may involve just a few molecules, play a role in the formation of larger aggregates. Transient oligomers may be particularly relevant for small peptides, which often show high conformational flexibility and a notably amphiphilic character. To obtain a good understanding of the aggregation behavior of a peptide, methods that can distinguish aggregates of different characters and sizes are of importance.

A wide range of approaches and techniques are applied for the detection of peptide aggregation and characterization of aggregates.^13,14^ Large fibrils or amorphous aggregates (of μm scale or larger) are detectable by optical microscopy or visual inspection, whereas aggregates in an intermediate size range (nm to μm) can be identified by, for instance, dynamic light scattering (DLS).^15^ Fluorescence spectroscopy (intrinsic or extrinsic) can be applied to investigate aggregates with distinct hydrophobic domains,^14,16^ while oligomers or protofibrils of sufficient stability can be studied by size exclusion chromatography^17,18^ or analytical ultracentrifugation.^13^ Unfortunately, approaches applied for investigating small peptide aggregates often involve changes in solution conditions, risk of binding to column materials, and so forth or require the addition of probe molecules, which may influence peptide self-assembly both qualitatively and quantitatively and thereby introduce a risk of inaccurate conclusions regarding the aggregation behavior.

In this work, ^1^H NMR spectroscopy and all-atom molecular dynamics (AA-MD) simulations were applied to investigate the aggregation behavior of a series of structurally similar peptides with previously known differences in aggregation propensity. ^1^H NMR spectroscopy is a nondestructive technique with great potential for in situ detection and investigation of peptide aggregation in solution, which does not require the addition of external probe molecules. When a peptide molecule takes part in an aggregate, it will experience changes in conformation, local chemical environment, and mobility. These changes can influence chemical shift, width, shape, and intensity of signals in an NMR spectrum. Evaluation of changes and differences in the appearance of ^1^H NMR spectra with variation in sample composition and conditions has been used for decades to investigate self-assembly of amphiphiles,^19−22^ and similar approaches have been applied in the investigation of peptide and protein aggregation in recent publications.^23−31^

All-atom molecular dynamics (AA-MD) simulations give insights into molecular-level events on short (nano- to microsecond) time scales. In particular, it is a useful tool for simulating interactions as molecules coming into contact. AA-MD simulations have been used to predict and reveal different aspects of aggregation behavior, for example, to address monomer addition versus cluster–cluster coalescence mechanisms,^32−34^ interactions between proteins and excipients,^35,36^ and to investigate aggregation pathways of the amyloid-β peptides—Aβ40 and Aβ42.^37^

**Figure 1:**
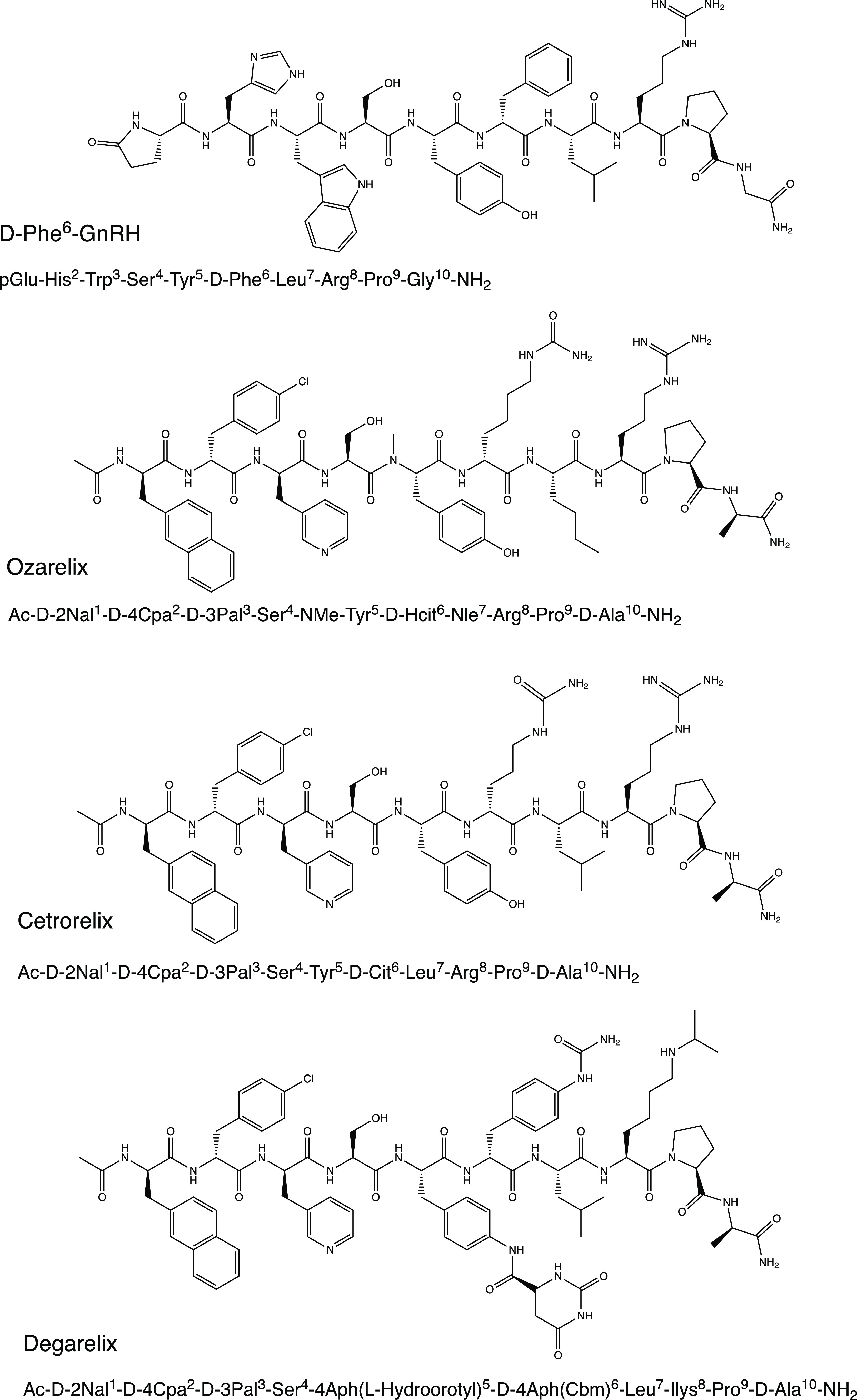
Molecular structures and amino acid sequences of the investigated peptides.

The experimental part of this work was performed with four structurally related decapeptides, which can be regarded as representative of a series of candidate drug substances in the late discovery/early development phase (see Figure 1), D-Phe^6^-GnRH, ozarelix, cetrorelix, and degarelix (acetate salts). These peptides are all analogues of gonadotropin-releasing hormone (GnRH) with previously known differences in aggregation propensity. One-dimensional ^1^H NMR in neat H_2_O was applied to study aggregation of these peptides in a systematic manner. The experiments were performed in regular water (H_2_O) rather than deuterated water (D_2_O), which is commonly used in NMR experiments, since the latter can have substantial influence on the behavior of molecules in solution, for example, self-assembly of amphiphiles,^38^ the flexibility of folded proteins,^39^ and may also affect the aggregation behavior of peptides. Furthermore, samples were prepared without buffer or other added electrolytes to avoid possible, complicating salt effects. Supportive NMR diffusometry measurements were performed on selected samples (in D_2_O). AA-MD simulations were performed with models of D-Phe^6^-GnRH (two different charge variants), ozarelix, and cetrorelix.

The aim of this work was to evaluate the applicability of the developed ^1^H NMR methodology and AA-MD simulations, individually and in combination, in developability assessments and formulation development of therapeutic peptides, and for investigation of solution behavior and aggregation propensity.

## 2 Materials and Methods

### 2.1 Materials

D-Phe^6^-GnRH acetate, ozarelix acetate, and cetrorelix acetate were prepared specifically for this study by Red Glead Discovery, Lund, Sweden, and degarelix acetate was a gift from Ferring Pharmaceuticals A/S. According to the respective providers, the free base contents of D-Phe^6^-GnRH, ozarelix, cetrorelix, and degarelix were 88, 95, 96, and 87%, respectively, whereas the molar ratios of acetate/acetic acid to peptide were 1.7, 1.0, 1.0, and 2.5. Aqueous solutions were prepared with water purified using a Milli-Q system. D_2_O (99.8%) used in the NMR diffusion experiments was obtained from Armar Isotopes, Germany.

### 2.2 Sample Preparation, Handling, and Characterization by Visual Inspection

^1^H NMR experiments were performed at peptide concentrations of 0.1, 0.5, 1, 2, 5, and 10 mM in 100% H_2_O. Peptide solutions were prepared in glass vials without pH adjustments, as the addition of concentrated acid or base results in local pH variations that might induce aggregation. Diffusion NMR experiments were performed at peptide concentrations of 3 and 10 mM in 100% D_2_O. Thus, the diffusion NMR results may not be perfectly representative of the situation in the samples in H_2_O, but general conclusions can still be drawn.

The samples were gently agitated, by manual swirling, until the solution appeared visually homogeneous and 450 μL of each sample was transferred to disposable 5 mm NMR tubes (Type 5TA 178 mm from Teknolab Sorbent, Sweden). Aliquots were pipetted slowly to reduce the risk of possible shear-induced aggregation. The time between sample preparation and the start of the NMR measurement was around 15 min. On selected samples, the approximate pH was assessed with pH indicator strips (Scharlau, pH 2.0–9.0, TP0209000S) after aliquots for the NMR samples were taken out.

The NMR tubes containing samples were visually inspected in a light box and compared to an NMR tube containing pure water to simplify identification of visually detectable aggregates.

### 2.3 ^1^H NMR—Instruments and Experimental Setup

NMR experiments were performed at 25 °C on either a Varian Unity Inova 500 MHz spectrometer equipped with a Z-spec DBG500-5EF 5 mm dual broadband gradient probe or a Bruker AVANCE III HD 500 MHz spectrometer equipped with a BBO probe, Bruker SMART probe. Spectra were recorded with an excitation sculpting sequence for solvent suppression.^40^ Experiments on the Varian spectrometer were run as soon as possible after sample preparation (typically within 15 min), after 24 h, and after 1 week with an excitation pulse width of 11.8 μs (corresponding to a 90° pulse), a spectral width of 8 kHz, an acquisition time of 2.048 s, and a recycle delay of 4.0 s. On the Varian, 300 scans were used on samples of 0.1–1 mM and 64 on 1–10 mM (1 mM was measured using both settings to bridge the concentration series and ensure comparability), which gave adequate signal for unaggregated samples. In the case of aggregation, reduction in signal strength is expected.

Experiments on the Bruker spectrometer were run as soon as possible after sample preparation (typically within 15 min), after 2 h, and after 48 h, with an excitation pulse width of 9.5 μs (corresponding to a 90° pulse), a spectral width of 6.6 kHz, and the collection of 512 scans. The acquisition time and recycle delay were kept the same within each concentration series but varied between the peptides to allow for appropriate decay of the FID. The combined settings for acquisition time and recycle delay were either 1.0 and 2.0 s or 2.9 and 0.1 s, respectively. To gain insights into possible changes in spectral appearance on a short time scale, two experiments with 4 scans were performed 10 min apart in connection to the initial measurement, and an additional 4 scan measurement was performed at all consecutive time points. Between NMR experiments, samples were left in an upright position on a gently rocking cradle at room temperature.

As no deuterated solvent was included in the samples, the ^1^H NMR experiments were performed without the application of a field-frequency lock. However, since the time scale of the experiments was relatively short and the spectrometers used did not show a notable drift, the omission of field-frequency lock did not have an appreciable impact on the quality of the recorded spectra. Shimming was performed by gradient shimming on the solvent (H_2_O) signal.

### 2.4 ^1^H NMR—Data Treatment

Each spectrum was phase- and baseline-corrected prior to integration in an ACD/Spectrus Processor (version 2021.1.1, Advanced Chemistry Development, Inc., Toronto, On, Canada, www.acdlabs.com 2021). The ppm scale was referenced to the rightmost peak from the leucine or nor-leucine side chains, for which the chemical shift was set to 0.7 ppm.

**Figure 2:**
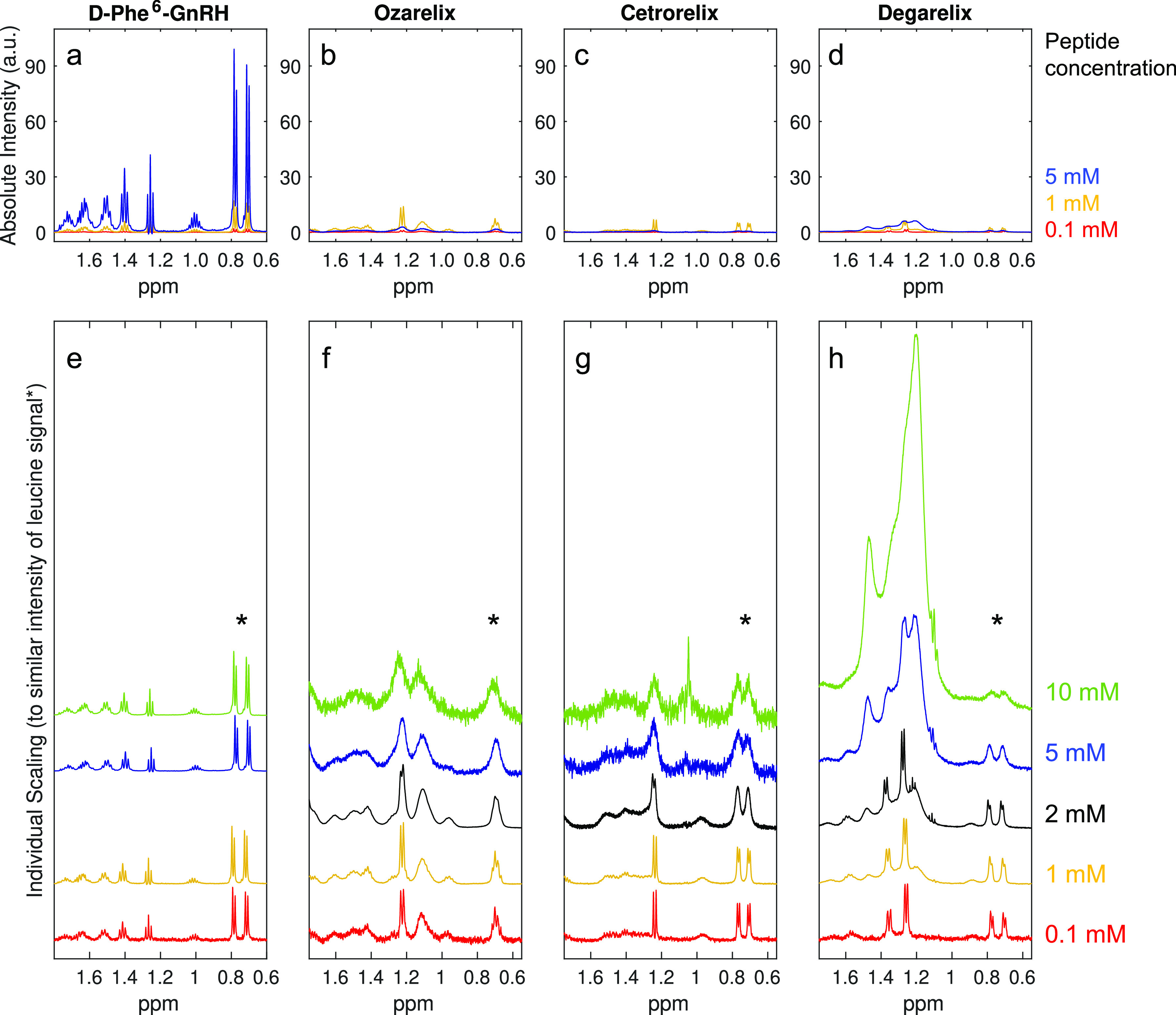
Partial ^1^H NMR spectra displaying the variation in spectral appearance with concentration. For a–d, the same intensity scale is used for all spectra to show changes in intensity. In e–h, spectra were individually scaled to yield similar intensity for the leucine/nor-leucine methyl peaks in all spectra.

**Figure 3:**
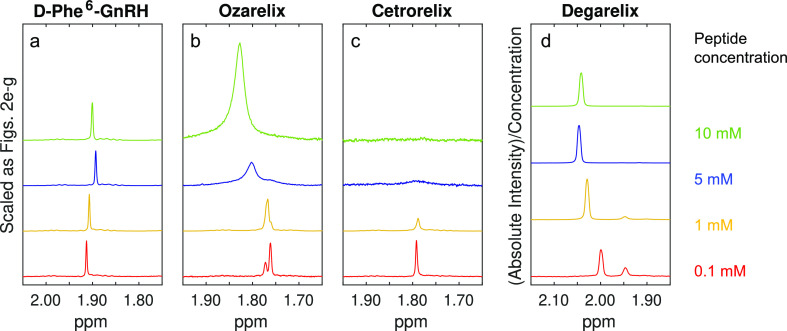
Partial ^1^H NMR spectra showing the development in appearance and chemical shift of the signals from the acetate counterion and the N-terminal acetyl group (see Section S3.3). The spectra in a–c are scaled as in Figure 2e–g, whereas degarelix spectra are normalized to concentration.

The absolute integral values (Int_C_), at each investigated concentration, for signals arising from the methyl groups of leucine (6H; for D-Phe^6^-GnRH, cetrorelix, and degarelix) or nor-leucine (3H; for ozarelix) were determined. This signal is a doublet of doublets or a multiplet at 0.7–0.8 ppm (Figure 2) and was selected as it did not show obvious overlap with other signals. From Int_C_, the normalized absolute integral value, NAI, was calculated as described in eq 1, where Int_C_min__ and *C*_min_ are the absolute integral value and the concentration for the lowest peptide concentration studied (0.1 mM), respectively. Additionally, Int_C_ of the signal from the acetate counterion (a singlet) were determined for all peptides. For ozarelix and cetrorelix, there was an overlap of the acetate signal and the signal from the N-terminal acetyl group (also a singlet; Figure 3); for these peptides, Int_C_ represents the combined integral area of both these peaks. Collected data were further processed to obtain a shape index, SI (defined in eq 2), and the concentration normalized absolute integral value, CNAI (defined in eq 3). The SI, based on the intensity to integral ratio, decreases with peak width, while CNAI diverts from unity as the signal disappears due to extensive broadening.
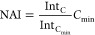
1
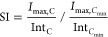
2
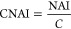
3*I*_max_ is the maximum absolute peak intensity value and *C* is the molar concentration.

### 2.5 NMR Diffusometry

NMR diffusion experiments were performed at 25 °C on a Bruker AVII-200 spectrometer equipped with a Bruker DIFF-25 gradient probe and a Bruker GREAT 1/40 gradient amplifier using a pulsed field gradient stimulated echo (PFG-STE) sequence. Additional details regarding the diffusion measurements, including relevant theory related to the interpretation of the results, are presented in Section S4.

### 2.6 AA-MD Simulations

Construction of D-Phe^6^-GnRH, cetrorelix, and ozarelix topologies was performed using the Charmm36 force field,^41,42^ with the non-natural residues in the peptides represented by parameters from the SwissSidechain database^43^ and incorporated into the peptides using the PyMol plugin provided on the SwissSidechain website. Degarelix was not included in the simulations due to parameterization challenges and because of the indications from NMR of a more complex aggregation behavior. In each AA-MD simulation, 20 peptides were placed in a cubic box with a side length of 15 nm, which gives a peptide concentration in the AA-MD simulations similar to the highest concentration used in the NMR experiments (i.e., 10 mM) and a setup with a good balance between simulation box size and computational efficiency. Simulations of cetrorelix and ozarelix were performed on peptide molecules with a total charge of +1 (on the arginine residue), whereas simulations of D-Phe^6^-GnRH were performed under two different conditions, with the histidine side chain being either neutral or charged (which gives total peptide charges of +1 or +2, respectively), resulting in a total of four simulated systems. Three independent simulations were performed for each system with the peptides initially placed randomly in the simulation box. The numbers of aggregates and free peptide monomers were calculated using an in-house Python code, with two peptide molecules considered to be in the same aggregate if any of their constituent atoms were found within a cutoff distance of 0.5 nm.^32^ The simulations were performed with chloride as the counterion, while the NMR experiments were performed on acetate salts of the respective peptides. The interactions between cetrorelix and chloride or acetate ions were compared by calculating the ion-peptide radial distribution functions and minor differences were found. While the choice of counterion can have an impact on long-term aggregation behavior, the observed differences in peptide–counterion interactions with the different counterions are not expected to be critical for the time scales and aggregation sizes studied. Additional details regarding the simulation setup and analysis protocols are presented in Section S6.

## 3 Results and Discussion

### 3.1 Macroscopic Behavior of Samples

Most samples, including all samples of D-Phe^6^-GnRH and degarelix, were visually clear over the investigated time span. Freshly prepared samples of cetrorelix at concentrations of 2 mM and above and ozarelix at concentrations of 5 mM and above showed a slight turbidity, which remained at all following time points of inspection (example photographs are shown in Figure S1). A faint turbidity also appeared within 2 h for samples of 1 mM cetrorelix and 2 mM ozarelix. The slight turbidity suggests the presence of aggregates or particles with sizes of a few hundred nanometers or larger. Furthermore, a slight increase in viscosity (identifiable by visual observation) with increasing concentration was noticed for all investigated peptides except for D-Phe^6^-GnRH.

The peptide samples showed pH in the range of ∼5.5 to ∼8.5, varying with peptide type, concentration, and time. At these pH values, all the investigated peptides residing in solution are expected to carry an average charge of +1 or somewhat higher. Additional details regarding sample pH and peptide charge are presented in Section S2.

### 3.2 NMR Results and Implications Thereof

This section begins with a brief presentation of how peptide aggregation is expected to influence ^1^H NMR spectra, followed by an overview of representative NMR data and a discussion on the significance of the results, with respect to the aggregation behavior of each peptide.

#### 3.2.1 Consequences of Aggregation on Appearance of ^1^H NMR Spectra

When a molecule, for example, a peptide, takes part in an aggregate, it is expected to experience changes in conformation, local chemical environment, and mobility compared to the situation when it is present as a monomer. The chemical shift (i.e., the position in the spectrum) of an NMR signal is influenced by molecular conformation and local chemical environment. On the other hand, the width and shape of NMR signals are influenced by molecular mobility and rate of reorientation (as a consequence of the influence of mobility on the spin–spin relaxation time, *T*_2_) and thereby by aggregate size. Significant signal broadening is typically observed with aggregates of sizes of tens to hundreds of nm, but line shape is also influenced by, for example, aggregate rigidity and geometry. Molecules residing in large aggregates (hundreds of nm or larger) become practically undetectable in liquid-state NMR (see Section S3.1 for additional comments on line width, *T*_2_, and aggregate size). Taken together, systematic evaluation of variations in the appearance of NMR spectra with concentration and time can provide insights into oligomerization and aggregation of peptides.

#### 3.2.2 Variation among ^1^H NMR Spectra with Concentration and Time

Partial ^1^H NMR spectra of samples of between 0.1 and 10 mM of the four investigated peptides are shown in Figure 2 (signals from aliphatic moieties of the peptide molecules) and Figure 3 (signals from acetate counterion and the N-terminal acetyl group). Additional details of the spectra of all samples and interpretations of these are presented in Figure S3.

**Figure 4:**
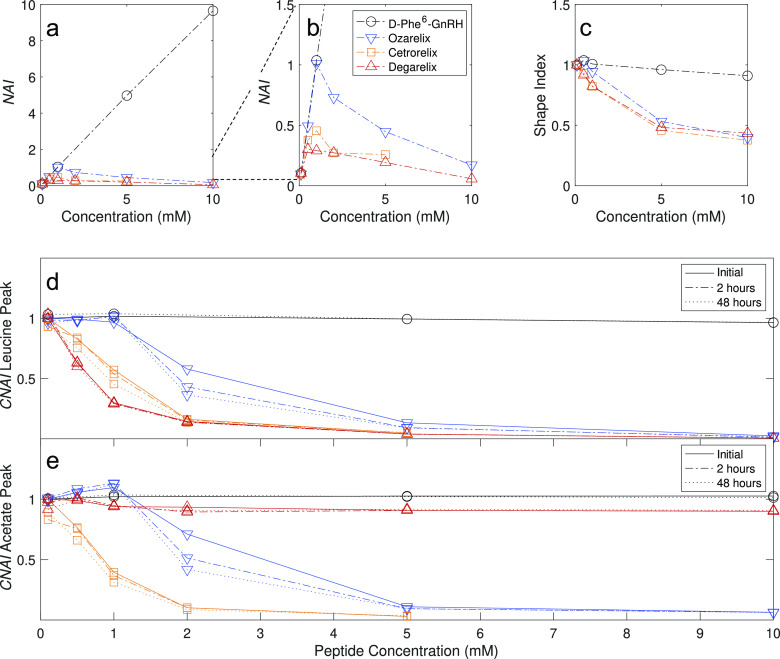
Different representations of the NMR data with concentration. (a–d) Data for the leucine or nor-leucine methyl groups (appearing at 0.7–0.8 ppm) and (e) data for the counterion acetate (appearing at 1.7–1.9 ppm). Panels (a,b) display NAI (see eq 1) for spectra recorded after 48 h. The SI (linked to peak broadening) for spectra recorded after 24 h is displayed in panel c (see eq 2). Panels (d,e) display CNAI (see eq 3).

For most samples, signal broadening (when observed) occurred to a similar degree for all signals in the respective NMR spectra and the relative intensities of signals within each spectrum were practically unchanged. Thus, the variation in intensity and shape of a single, well-separated signal is largely representative of the whole spectrum (the main exception is the group of broad signals between 1.2 and 1.6 ppm observed for samples of degarelix at 1 mM or higher, see Figure 2h and discussion below). The variation of integrals and intensities of such a signal with concentration and time can be used for straightforward comparison of key trends in the NMR results. Figure 4a–d is derived from the signals of the methyl groups of amino acid residue 7 (leucine or nor-leucine). Figure 4a,b shows the variation in NAI (defined in eq. 1) with concentration. In this representation, deviation from a linear increase in NAI suggests aggregation. Figure 4c shows the variation in SI (as defined in eq. 2) with concentration, where lower SI represents broader signals. Figure 4d presents the CNAI (defined in eq 3) at different concentrations and time points. Here, a decrease in CNAI with concentration indicates onset of aggregation or an increase in the aggregated fraction (if aggregates are present already at the lowest investigated concentration). Figure 4e shows the corresponding CNAI plot for the signals from the acetate counterion (or in the cases where signals from acetate and N-terminal acetyl group are overlapping, a sum of both integrals).

Most samples showed no notable changes in CNAI and line shape over the investigated time span. The main exceptions were observed for cetrorelix and ozarelix at 1 and 2 mM, which showed substantial decrease in CNAI over time (Figure 4d). Additional comments on changes over time are found in Section S3.4.

#### 3.2.3 D-Phe^6^-GnRH

The spectra of D-Phe^6^-GnRH show an essentially linear increase in absolute integral with increasing concentration (Figure 4a,b,d) and a practically unchanged general appearance, with narrow signals throughout the investigated concentration and time ranges (Figures 2e and 4c). Furthermore, the behavior of the acetate counterion signal parallels that of the signals from the peptide itself (Figures 3a and 4e). These observations suggest that D-Phe^6^-GnRH is predominantly present as individually dissolved monomers. The conclusion that self-assembly of D-Phe^6^-GnRH is limited (or absent) is further supported by results from NMR diffusion measurements. The samples of 3 or 10 mM D-Phe^6^-GnRH (in D_2_O) both showed very similar self-diffusion coefficients, consistent with a majority (or all) of the peptide being present as individual molecules in solution in the whole investigated concentration range (presence of a minor fraction of transient oligomers cannot be excluded based on the collected NMR diffusion results, see Section S4 for additional comments).

#### 3.2.4 Ozarelix and Cetrorelix

Ozarelix and cetrorelix both show maxima in intensity and absolute integrals between 0.5 and 2 mM (Figures 2b,c and 4a,b,d), as well as a similar degree of progressive, spectrum-wide broadening between 2 and 10 mM (Figures 2f,g and 4c). Peak broadening and loss of signal is attributable to aggregation, and based on the plots of CNAI and SI versus concentration, the NMR results demonstrate the occurrence of substantial aggregation from at least ∼0.1 mM and above for cetrorelix and above ∼1 mM for ozarelix (Figure 4c,d). The fact that ozarelix shows a CNAI of ∼1 up to the 1 mM data point suggest that it is predominantly present as monomers and/or resides in transient oligomers up to this concentration. For cetrorelix, where the CNAI decreases with concentration immediately above 0.1 mM (as well as with time), large aggregates may also be present at the lowest concentration investigated in this study (because of normalization to the integral for the 0.1 mM sample) and the concentration for onset of aggregation can thus not be determined.

For both ozarelix and cetrorelix, signals from the acetate counterion (a singlet) show considerable overlap with the signal from the N-terminal acetyl group (also a singlet; Figure 3b,c and additional comments in Section S3.3). However, it is still clear that the signals from acetate show broadening and signal loss similar to what is observed for the peptide itself (Figures 3b,c and 4e). This shows that the acetate counterions are predominantly included in the peptide aggregates formed.

The onset of effective signal loss at lower concentrations for cetrorelix than ozarelix (Figure 4d,e) is consistent with the observation of visually detectable turbidity at lower concentration for cetrorelix than ozarelix (at 2 and 5 mM, respectively, at the initial time point). Furthermore, the reductions in absolute integral over time for the samples of 1 mM cetrorelix or 2 mM ozarelix (Figure 4d) are correlated with the appearance of turbidity. However, the NMR results clearly show substantial aggregation at concentrations well below the concentrations where aggregation is detectable by turbidity. Evaluation of ozarelix and cetrorelix samples in NMR diffusion measurements (at 3 and 10 mM in D_2_O) was not possible due to the presence of large aggregates, that is, appropriate signal was not observed, see Section S4. The absence, at higher concentrations, of sharp signals from peptide and counterions in solution suggests a very low peptide solubility, that is, a low fraction of nonaggregated peptide and/or fast exchange between individually dissolved monomers or oligomers and molecule aggregates (although the latter is considered less likely for a peptide with known tendencies to form β-sheets).^44,45^

#### 3.2.5 Degarelix

Overall, the ^1^H NMR spectra of degarelix show a similar development with concentration as the spectra of ozarelix and cetrorelix. However, the results for degarelix differ in two important respects: (I) in addition to the general signal broadening with increasing concentration, a distinct group of broad peaks appear in the range between 1.2 and 1.6 ppm at concentrations of 1 mM or higher (Figure 2h), and (II) the absolute integral of the acetate counterion signal, which remains narrow over the investigated concentration range, increases linearly with the nominal peptide concentration (Figures 3d and 4e).

The relative intensity of the group of peaks between 1.2 and 1.6 ppm increases with concentration (Figure 2h). Furthermore, the signals in this range appear to be distinctly different from the other signals in the same spectra (with respect to chemical shift and line shape) and are practically invariant in shape at all concentrations where they are observed. These findings suggest that they arise from aggregates involving a fraction of peptide distinct from that resulting in practically complete signal loss, which show slow exchange with monomers and/or small transient oligomers in solution. This is also supported by results from the NMR diffusometry experiments (at 3 and 10 mM in D_2_O), which suggest the presence of distinct fractions of intermediately sized aggregates ranging from single molecules and/or small oligomers up to aggregates of at least tens of molecules (see Section S4 for additional comments).

The fact that the signal from the acetate counterion parallels the nominal peptide concentration reveals that the counterions largely remain in solution when degarelix self-assemble into larger structures. In turn, this suggests that degarelix resides in aggregates predominantly in its uncharged form. The idea that the acetate ions are largely free in solution is supported by the NMR diffusion measurements, which show that the self-diffusion coefficient of the acetate ions in the degarelix samples is similar to that recorded for acetate in the D-Phe^6^-GnRH samples.

To summarize, degarelix appears to reside in at least three categories of aggregates: free monomers/small oligomers, intermediately sized aggregates (which give rise to the group of broad NMR signals between 1.2 and 1.6 ppm), and large aggregates (which are largely undetected in the NMR spectra). The large aggregates in degarelix samples do not result in visually detectable turbidity. This indicates that the aggregates formed differ in size and/or structure from those of ozarelix and cetrorelix.

### 3.3 AA-MD Simulations

#### 3.3.1 Molecular Aggregation Pathways

**Figure 5:**
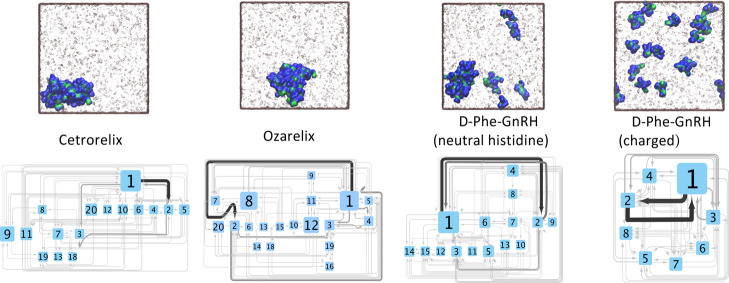
Top: Simulation snapshots (at 500 ns) of cetrorelix, ozarelix, and D-Phe^6^-GnRH. Peptide backbone and side chain atoms are colored green and blue, respectively. Bottom: Peptide aggregation transition networks. Numbers on each node represent the number of molecules in an aggregate, and the size of the nodes is proportional to the number of aggregates with that particular size. Arrow thickness is proportional to the number of transitions between that pair of nodes.

The aggregation pathways, as observed in the simulations, are shown in Figure 5. It can be seen in snapshots of the peptides after 500 ns (Figure 5, top) and from aggregation transition networks (Figure 5, bottom) that both cetrorelix and ozarelix monomers coalesce over time into aggregates that include all 20 peptide molecules present in the simulation box. For D-Phe^6^-GnRH, the aggregate size is much smaller, and the maximum aggregate size found in the simulations was *n* = 15 and *n* = 8 with uncharged (peptide net charge of +1) and charged histidine (peptide net charge of +2), respectively.

It is evident for all the studied peptides that aggregation mainly starts with monomers forming dimers and trimers. These then grow either by monomer addition or by being associated with other small aggregates, resulting in the formation of larger aggregates. For cetrorelix and ozarelix, these aggregates then coalesce, eventually leading to the formation of aggregates containing all 20 peptide molecules. In the simulations of D-Phe^6^-GnRH(His+), dimers were seen to dissociate following their formation, and there was practically no coalescence of medium-sized aggregates.

#### 3.3.2 Molecular Aggregation Dynamics

**Figure 6:**
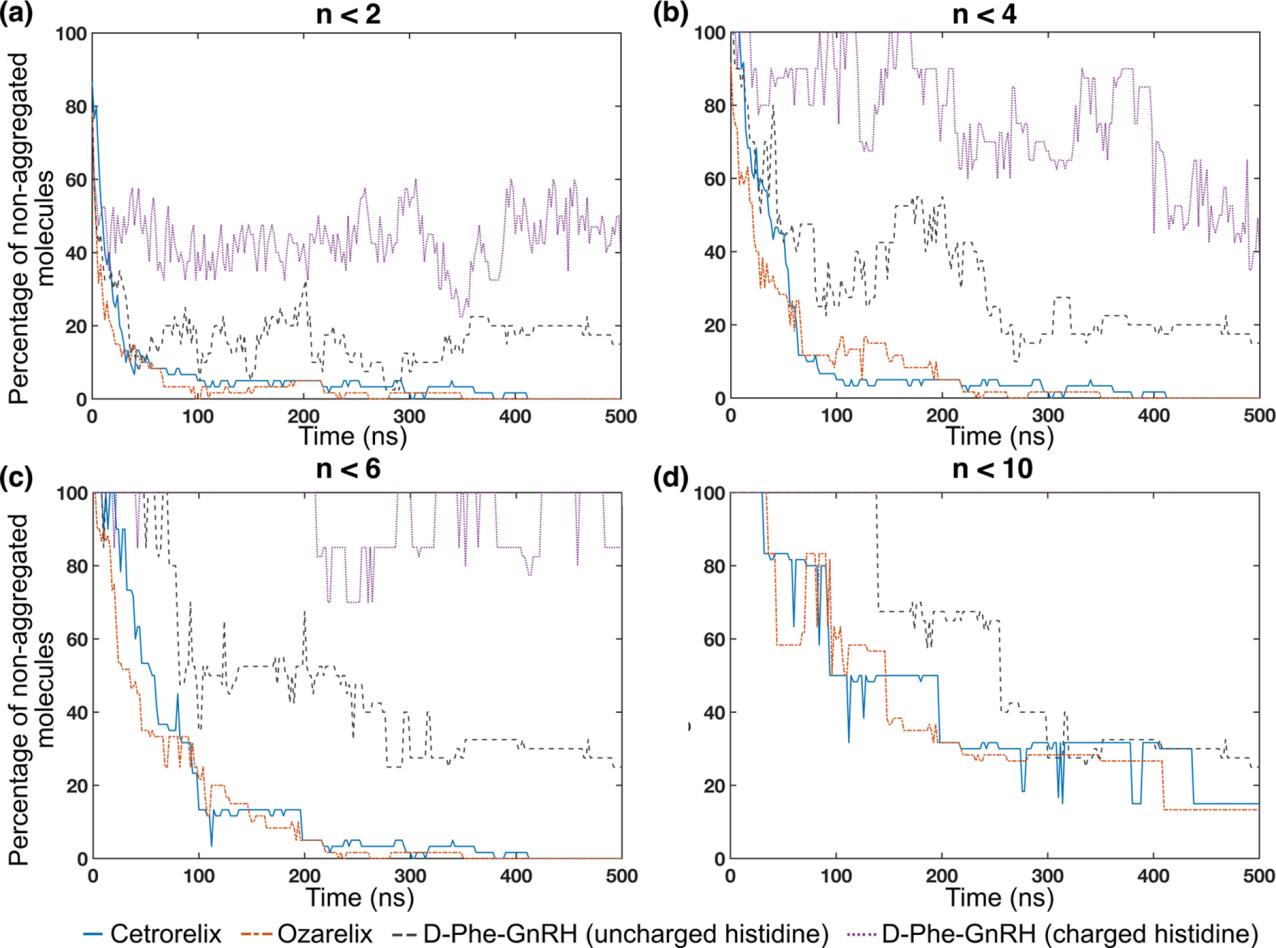
Evolution of the percentage of nonaggregated peptides during AA-MD simulations at aggregate cutoff sizes *n* = 2, 4, 6, and 10, shown in panels (a–d), respectively.

When evaluating the simulation data, the number of peptide molecules needed to define an aggregate can be set at different levels, by varying the aggregation cutoff size. For instance, with a cutoff size *n* = 2, only individual peptide monomers are considered nonaggregated, compared to *n* = 10, where peptide monomers and oligomers consisting of less than 10 peptide molecules are considered nonaggregated. Evaluation of different cutoff values allows us to further investigate the dynamics of the peptide aggregation process. The percentages of nonaggregated peptide molecules were calculated by considering four aggregate cutoff sizes (*n* = 2, 4, 6, and 10) as shown in Figure 6. The fraction of nonaggregated peptides for cetrorelix and ozarelix is greatly reduced during the simulation (500 ns) for all cutoff sizes. For D-Phe^6^-GnRH (both uncharged histidine and His+), the nonaggregated fraction, at the end of the simulation, increases with aggregate cutoff size. With *n* = 2, the number of free monomers for cetrorelix and ozarelix is rapidly reduced, reflecting an initial period where the system is dominated by the diffusion of peptide monomers. This phenomenon has been observed in other simulation studies of peptide aggregation.^34,46,47^ Hence, there is a very low percentage of free monomers for much of the simulation, eventually reaching zero at 400 ns. For D-Phe^6^-GnRH with uncharged histidine, the fraction of free monomers is similarly reduced early in the simulations but then stabilizes at around 20% of free monomer, while D-Phe^6^-GnRH with charged histidine (His+) stabilizes at about 40%. Thus, the presence of the charged histidine increases the percentage of free monomers for D-Phe^6^-GnRH compared to the D-Phe^6^-GnRH with uncharged histidine. At higher cutoffs, cetrorelix and ozarelix showed a similar behavior, and even with *n* = 10, the amount of nonaggregated peptide was low at 500 ns. In contrast, at *n* = 10, all D-Phe^6^-GnRH(His+) molecules are present either as monomers or as smaller oligomers (in Figure 5, the maximum aggregate size is *n* = 8). The formation of medium-sized aggregates (at least up to *n* = 6 molecules) appears to be a more rapid process for cetrorelix and ozarelix than for D-Phe^6^-GnRH.

**Figure 7:**
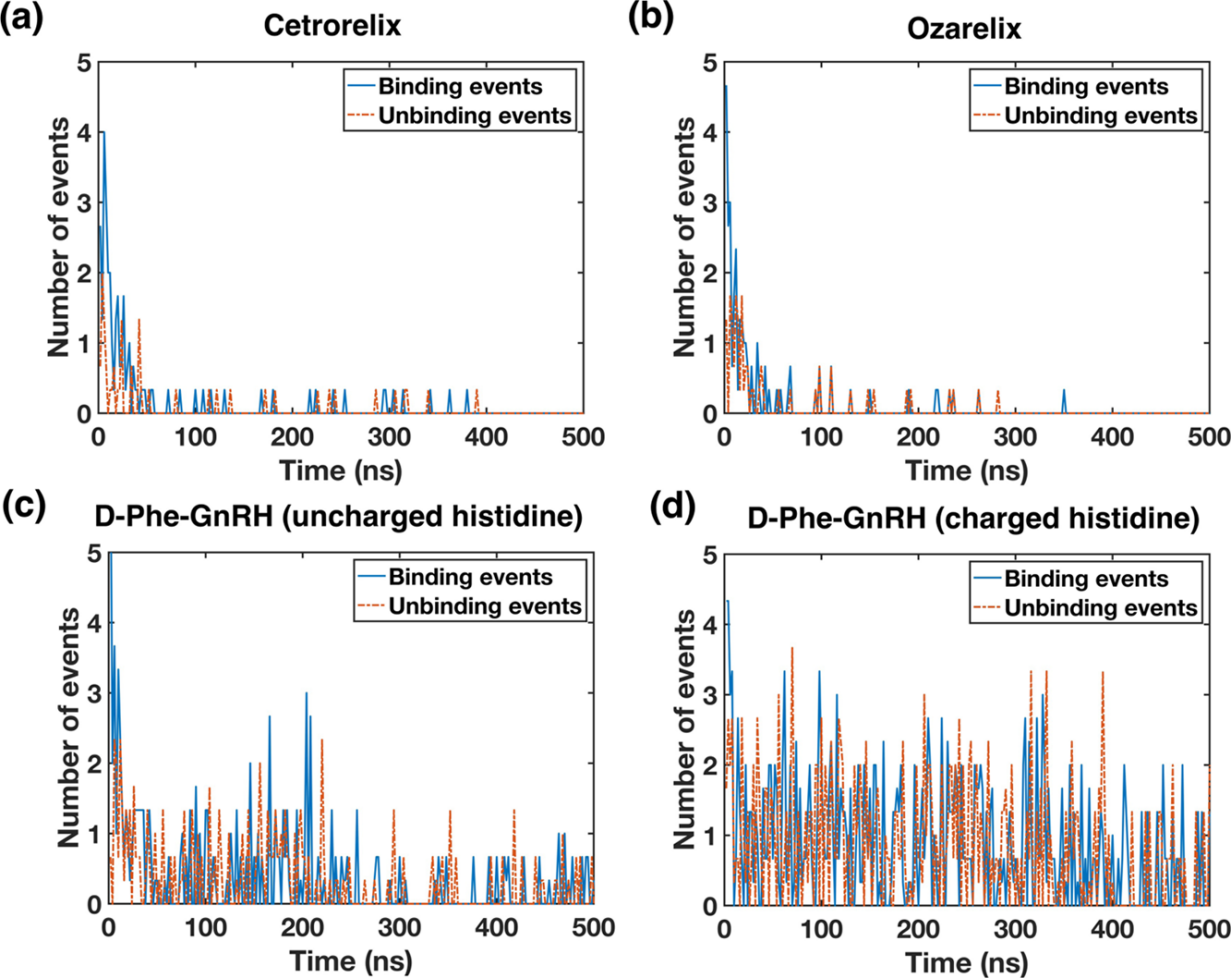
Number of binding and unbinding events during the simulation period.

To give additional insights into aggregation dynamics, peptide–peptide binding and unbinding events were quantified from the simulation data. After about 50 ns, binding or unbinding rarely occurred for cetrorelix and ozarelix (Figure 7a,b). This is consistent with rapid onset of aggregation and in contrast to the result for both D-Phe^6^-GnRH configurations (Figure 7c,d). Assemblies of both D-Phe^6^-GnRH configurations, particularly D-Phe^6^-GnRH(His+), are very dynamic, with binding or unbinding occurring at the same level throughout most of the simulation, indicating that oligomers are formed and dissociated throughout the simulation.

The collision acceptance probability, CAP, was calculated from the number of binding and unbinding events according to eq S5. A CAP value close to one is taken to indicate an aggregation prone peptide. While simulations are not directly comparable with experiments, due to the difficulties of tracking single-molecule binding and unbinding experimentally, CAP values provide an intuitive way to rank peptides in terms of aggregation propensity and have previously been used for peptides both experimentally and in simulations.^34^ CAP values are also independent of the time period for which a peptide monomer stays bound or unbound, in contrast to association and disassociation constants. Calculated CAP values for the simulated peptides are found in Table S4. Based on these values, the following rank order of aggregation propensity can be proposed: cetrorelix > ozarelix > D-Phe^6^-GnRH (uncharged histidine) > D-Phe^6^-GnRH(His+).

#### 3.3.3 Molecular Interactions within the Aggregates

**Figure 8:**
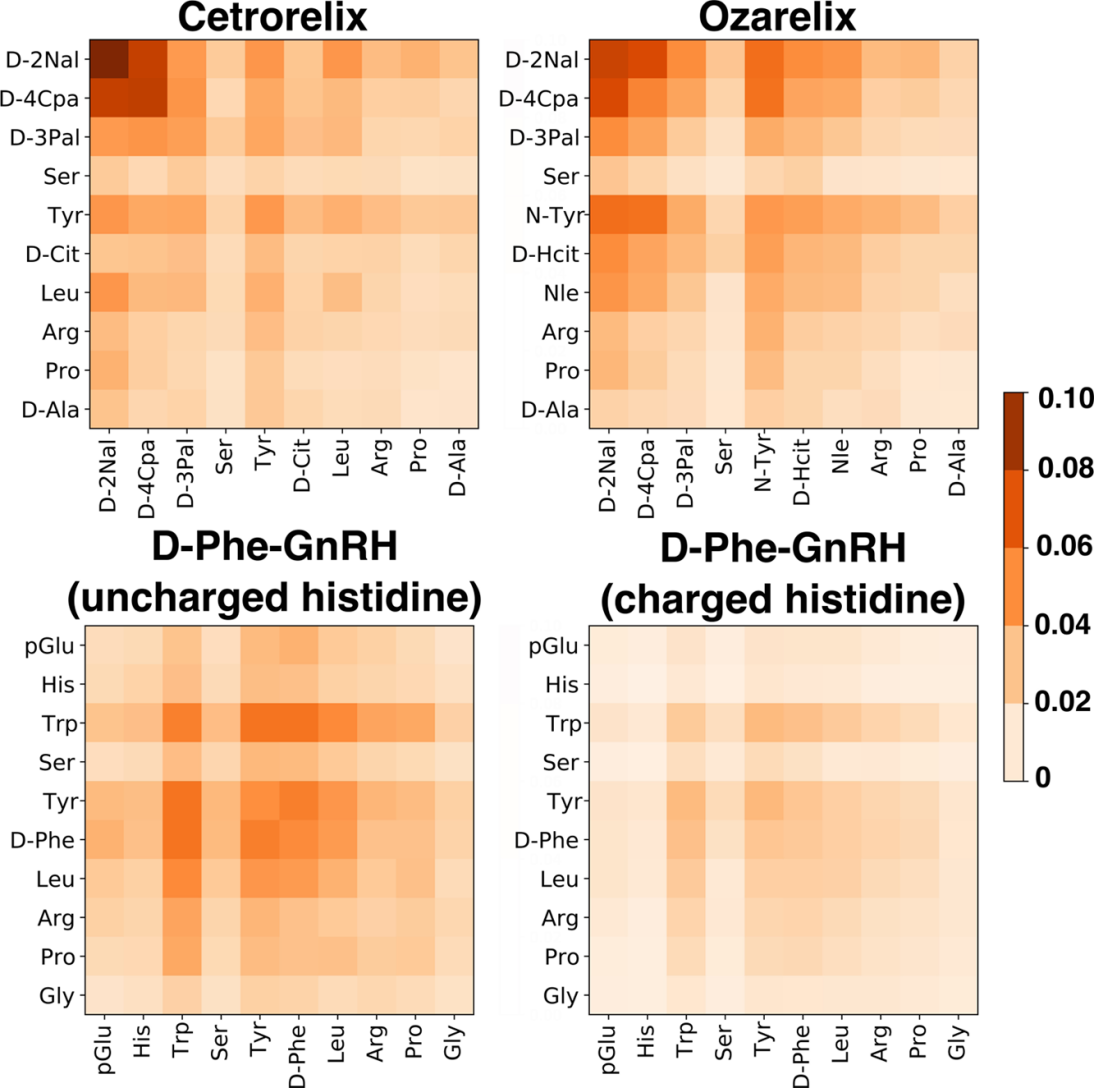
Average interpeptide residue–residue contact frequencies, with contacts normalized by the number of cetrorelix contacts.

Peptide–peptide contacts, including hydrogen-binding patterns, were analyzed to gain further molecular insights into possible predominant peptide–peptide interactions within the aggregates. The results (Figure 8) indicate that for both cetrorelix and ozarelix, residues near the N-terminal (*N*-acetyl-d-(β-naphthyl)alanine (D-2Nal), d-(4-chloro)phenylalanine (D-4Cpa), d-(2-pyridyl)alanine (D-3Pal), and tyrosine (Tyr) or *N*-methyl tyrosine (N-Tyr) play a dominant role among the intermolecular contacts. This seems to contribute to differentiating the overall aggregation propensity of these peptides. In contrast, although D-Phe^6^-GnRH (neutral histidine and His+) shows fewer peptide–peptide interactions, overall, peptide–peptide interactions are dominated by the hydrophobic residues (tryptophan, tyrosine, and phenylalanine). Electrostatic peptide–peptide repulsion between charged histidine side chains is also evident for D-Phe^6^-GnRH(His+).

Hydrogen bonds are formed between peptide molecules and between peptide molecules and water (Figure S7). The least aggregation-prone peptide [D-Phe^6^-GnRH(His+)] forms the largest number of peptide–water hydrogen bonds, while cetrorelix has the highest number of peptide–peptide hydrogen bonds. The simulations indicate intermolecular hydrogen bonds particularly for the tyrosine moiety in cetrorelix (Figure S8).

Finally, to complement understanding of the molecular interactions that might cause the variations in aggregation behavior among the peptides, the exposure of the peptide amino acids to the solvent was characterized by assessing the hydrophobic solvent-accessible surface area (hSASA, summarized in Table S5). Again, a distinction between cetrorelix/ozarelix and D-Phe^6^-GnRH can be observed, already for monomers and dimers. The average hSASA for cetrorelix and ozarelix is similar, while it is roughly 20% lower for D-Phe^6^-GnRH. The relatively lower hSASA values of D-Phe^6^-GnRH might thus in part explain the reduced aggregation tendency of this peptide. The hSASA value does not allow for a clear distinction between the two D-Phe^6^-GnRH systems; the values are within the standard deviation of each other, which presumably reflects the delicate balance between electrostatics, hydrophobic interactions, and conformational flexibility.

### 3.4 Information on Peptide Aggregation Provided by the Applied Methodologies

#### 3.4.1 Aggregation Propensity Rank Order

The peptide aggregation propensity rank order can be assessed with both the NMR and AA-MD methodologies evaluated in this work. The peptides investigated by AA-MD simulations were shown to give the same rank order with respect to the general aggregation propensity as in NMR; cetrorelix > ozarelix > D-Phe^6^-GnRH. This rank order is also consistent with results from a previous fluorescence spectroscopy investigation of the same three peptides (in 10 mM ammonium acetate buffer at pH 7.0). The fluorescence study showed critical peptide aggregation concentrations (CAC) of 0.04 and 0.17 mg/mL (∼0.03 and ∼0.1 mM) for cetrorelix and ozarelix, respectively, whereas no distinct aggregation concentration was identified for D-Phe^6^-GnRH at concentrations up to 10 mg/mL (8 mM).^16^ In the current NMR study, peptide concentrations above the peptide CAC (as determined with fluorescence) were investigated; still, the same peptide aggregation rank order was observed. The NMR results suggest that although there appears to be notable differences in the character of the aggregates formed, the general aggregation propensity of degarelix is similar to that of cetrorelix.

The predicted peptide aggregation rank order, among a series of related peptides, can be described by comparison of single numbers through calculation of the CAP from the number of binding and unbinding events in the AA-MD simulations. The peptide aggregation rank order from NMR is visualized through the CNAI expressed as a function of peptide concentration (Figure 4d).

#### 3.4.2 Coexistence of Different Aggregate Types

The NMR results on degarelix illustrate that it can be possible to identify the presence of coexisting aggregate types. For different aggregates to give distinct signals, the rate of exchange of peptide molecules between aggregates of different types needs to be relatively slow; the exact time scale depends on differences in chemical shifts and/or line shapes of signals in the spectra of the individual aggregate types.

In some instances, the fact that large aggregates may not give rise to a detectable NMR signal can be an obvious drawback. For instance, the presence of a small fraction of large aggregates can easily remain undetected in an NMR experiment. On the other hand, this also means that NMR experiments can allow for detection of variations or changes (or lack thereof) in a fraction of smaller aggregates that, in the presence of coexisting large aggregates, can be difficult to capture using alternative techniques (e.g., by DLS, for which results are weighted by larger particles). Results from a previous study, where ^1^H NMR was evaluated as a potential tool for quality control of the lyophilized peptide drug product, exemplify such a situation. For a series of samples of FIRMAGON (degarelix) drug product reconstituted in D_2_O, NMR spectra remained practically unchanged over time while an increase in the fraction of large aggregates was detected by DLS.^30^

The fact that signal from a small fraction of large aggregates is effectively filtered out in ^1^H NMR can be useful in developability assessment of drug substance batches containing significant levels of impurities (which is commonly the case for early development batches). If impurities show higher aggregation propensity than the main component, methods sensitive to the presence of a small fraction of large aggregates (such as DLS) may result in inaccurate conclusions regarding aggregation propensity.

#### 3.4.3 Influence of Different Structural Elements on Peptide Aggregation Propensity

The AA-MD simulations provide information regarding the influence of different structural elements on peptide aggregation propensity. A key conclusion from the contact map in Figure 8 is that close interactions among side chains of aromatic amino acid residues are important points of interaction for all peptides investigated. Based on the contact maps, the main point of attractive interaction among the peptide molecules is the *N*-acetyl-d-(β-naphthyl)alanine (D-2Nal) residue that is present at the N-terminal in ozarelix, cetrorelix, and degarelix, which all show extensive aggregation at low concentration, but is absent in the structure of D-Phe^6^-GnRH. This finding is consistent with a general notion that hydrophobic interaction is a key driver in peptide aggregation and is specifically supported by results from intrinsic fluorescence experiments, where fluorescence shifts were observed for D-2Nal as aggregation was identified.^16^ It is expected that the position of hydrophobic amino acids in the peptide sequence is a factor of importance for the degree of hydrophobic interactions among peptide molecules.^8,48,49^ A location close to an end of the peptide chain can likely promote attractive interaction (less steric hindrance and higher exposure to surrounding), whereas proximity to a charged amino acid can hamper close interaction (opposing electrostatic repulsion).

The importance of certain intermolecular hydrogen bonds suggested by the simulations, particularly for the tyrosine residue in cetrorelix (Figure S8), also correlates with findings from the previous fluorescence study.^16^

#### 3.4.4 Fate of Counterions in Peptide Aggregation

The different results on the acetate counterions for the three aggregating peptides show that ^1^H NMR can be applied to distinguish inclusion (as for ozarelix and cetrorelix) from exclusion (as for degarelix) of organic counterions in peptide aggregates formed. Inclusion or exclusion of counterions in peptide aggregates can be difficult to capture with other techniques and is a, to our knowledge, rarely discussed but potentially important aspect of peptide aggregation. The fate of the counterion on aggregation of a certain peptide is potentially dependent on the type of counterion (specific ion effects) and on the solution conditions (pH, type(s), and concentration(s) of cosolutes) and may be associated with fundamental differences in the structure and other properties of the aggregates formed. ^1^H NMR can thus be useful in counterion-screening studies aiming to tune the properties of a peptide in aqueous formulation.

#### 3.4.5 Effect of Solution Conditions on Peptide Aggregation

The herein-used NMR methodology is readily applicable in investigations on the impact of solution conditions (type and concentration of excipients, pH, etc.) on peptide aggregation and can thereby be valuable in, for example, formulation screening studies. The possibility to use ^1^H NMR for evaluating the impact of excipients on peptide aggregation is exemplified by results from the aforementioned study,^30^ where it was shown that buffer or background salt strongly enhance aggregation of degarelix (observed as reduction in total detectable signal and a decrease in the fraction of sharp signals). It should be kept in mind that organic cosolutes give NMR signals that may be superimposed on certain signals from the peptide. However, study of variations in general features of the peptide spectrum, such as variation in line broadening or the intensity of the detectable signal in the presence of organic solutes, can often still be possible without major problems.

The impact of solution conditions on aggregation can to some extent be investigated also in simulations. Effects of varying pH can be addressed by performing simulations on models with different protonation states. In the simulations of D-Phe^6^-GnRH, the aggregation propensity was found to be lower with charged histidine (i.e., with a total peptide charge of +2) than with uncharged histidine (total peptide charge of +1), which is consistent with an expected importance of electrostatic repulsion for a reduction in aggregation propensity. This result is also well in line with consequences of variation in charge observed experimentally for triptorelin, another GnRH analogue with an amino acid sequence similar to that of D-Phe^6^-GnRH.^50^ However, irrespective of the aggregation state, D-Phe^6^-GnRH shows lower aggregation propensity than both ozarelix and cetrorelix, which both carry a total charge of +1 in the simulations, illustrating the importance of the specific amino acid sequence for controlling aggregation propensity.

#### 3.4.6 Variation in the Extent of Aggregation over Time

The NMR results on ozarelix and cetrorelix show that ^1^H NMR can be applied to study variations in the extent of aggregation over time. This aspect is also exemplified by previously published results where variations in the degree of aggregation of degarelix over time were identified in the presence of buffer or background salt,^30^ which illustrates a useful application in excipient screening studies.

### 3.5 Application of NMR and AA-MD Simulation in Developability Assessments and Early Formulation Development of Therapeutic Peptide Formulations

At early stages of development, the amount of peptide available is usually very limited and nondestructive methods, such as NMR, and reliable simulation tools for predicting aggregation propensity are desirable.

Results presented above demonstrate that the herein-developed NMR methodology, which can be applied using conventional experimental procedures on standard ^1^H NMR spectrometers, is useful for detection of peptide aggregation and differentiation of structurally similar peptides with respect to aggregation propensity and behavior. For NMR studies in general, the required amount of the sample and the experimental time depend on several aspects, including the peptide concentration range of interest, the molecular structure of the peptide (which influences, e.g., the signal splitting patterns, the degree of signal overlap, and the number of protons contributing to a certain signal), and the sensitivity of the spectrometer used. Using lower-volume NMR tubes, lower sample amount is sufficient. However, a reduction in sample amount will typically increase the required experimental time and thereby limit the temporal resolution for investigation of peptide aggregation. NMR is typically not suitable for monitoring of fast aggregation processes, as the required experimental time is usually minutes or longer but is well-suited for the investigation of longer-term physical stability.

In contrast to more advanced NMR techniques,^51^ the herein-applied ^1^H NMR experiments cannot reveal details regarding aggregate structures but can, as is illustrated by the results presented above, readily identify the occurrence of extensive aggregation (by substantial line broadening or loss of signal) and qualitative differences in the aggregation behavior among peptides. Plots as those presented in Figure 4 allow a straightforward comparison of ^1^H NMR spectra for a series of peptides and evaluation of differences in their aggregation behavior. Furthermore, the results from this study illustrate that ^1^H NMR can be used to study aspects of peptide aggregation that can be difficult to capture using alternative techniques, such as the presence of different populations of aggregates (e.g., the intermediate aggregates observed for degarelix) and the inclusion or exclusion of counterions in aggregates. Taken together, the present work shows that ^1^H NMR in neat H_2_O constitutes a powerful tool for the investigation and classification of peptide aggregation.

In silico methods have become a growingly important tool in the drug discovery phase of design of new drug substances.^52^ There is a strong interest in obtaining similar tools for the drug development phase. The results from this work show that AA-MD simulations can give important information for early development of peptide drugs. It was found that there was a good correlation between the experimental and AA-MD simulation results. A drawback for AA-MD simulations is that setup and execution can be slow, limiting the time and length scales that can be accessed in studies. This can in part be alleviated by enhanced sampling techniques but will be particularly obvious when low concentrations of molecules are needed, in which case a balance must be struck between the size of the simulation box and the number of included molecules. This makes it difficult to study, for example, low concentrations of peptides. Increasing computing power and continued development of algorithms will improve this.

An advantage of simulations is that these can give indications of the fast dynamics in the system. In this work, this was seen in both the peptide aggregation transition networks and the frequency of binding and unbinding events. It could, for instance, be shown that there was a clear difference in the numbers of binding and unbinding events between the peptides that have been shown experimentally to have a large propensity to aggregate and those that have low aggregation propensity. Another strength of simulations is that they give detailed molecular information about the aggregates, allowing for hypotheses about specific interactions in and between the peptide molecules, in this work illustrated by the fact that it was possible to identify the amino acids that are most likely involved in interactions within the aggregates. Another example of how simulations can provide information on the molecular level is the study of the charged/uncharged histidine residue that provided information on possible pH effects on the peptide. Taken together, results from simulations complement experimental results and help to bring an increased understanding of the studied systems.

## 4 Conclusions

The herein-applied NMR methodology, where ^1^H NMR spectroscopy in H_2_O is combined with appropriate treatment of data, allows investigation of peptide aggregation with concentration, solution conditions, and time. Results show that ^1^H NMR can be used to detect the presence of coexisting classes of aggregates and inclusion or exclusion of counterions in peptide aggregates formed. The results from the AA-MD simulations provide information on the aggregation behavior on short time scales and information on the amino acid residues involved in peptide–peptide interactions within aggregates. The results from NMR and AA-MD simulations correlate well with respect to aggregation propensity for D-Phe^6^-GnRH, ozarelix, and cetrorelix. Taken together, the results from this study illustrate that both ^1^H NMR and AA-MD simulations can be useful tools in evaluation of the aggregation propensity of therapeutic peptides during developability assessment and early formulation development.

## Supporting Information Available

Selected photographs of samples for visual appearance, comments on pH and charge, supplementary ^1^H NMR spectra, NMR diffusion results and comments, additional comments on AA-MD simulation analysis, calculated collision acceptance probability, and supplementary figures on contact analysis (PDF)
