# Supplementary material for: Aggregation Behavior of Structurally Similar Therapeutic Peptides Investigated by 1H NMR and All-Atom Molecular Dynamics Simulations
Source: Mol Pharm. 2022 Feb 1;19(3):904–17. doi: 10.1021/acs.molpharmaceut.1c00883 (PMC8905580; doi:10.1021/acs.molpharmaceut.1c00883)
Supplement: Supplementary file 1 — mp1c00883_si_001.pdf [file mp1c00883_si_001.pdf]

Supporting information for

Aggregation behavior of structurally similar therapeutic peptides investigated by ^{1}H NMR and all-atom molecular dynamics simulations

Johanna Hjalte[1*], Shakhawath Hossain[2], Andreas Hugerth[3], Helen Sjögren[3], Marie Wahlgren[1], Per Larsson[2], Dan Lundberg[4#]

[1] Food Technology Engineering and Nutrition, Lund University, Box 124, 221 00 Lund, Sweden.

[2] Department of Pharmacy, Drug Delivery, Uppsala University, Box 580, 751 23 Uppsala, Sweden.

[3]Ferring Pharmaceuticals A/S, Amager Strandvej 405, 2770 Kastrup, Denmark.

[4]CR Competence AB, Center for Chemistry and Chemical Engineering, Box 124, 221 00, Lund, Sweden.

[#]Present address: Advanced Drug Delivery, Pharmaceutical Sciences, R&D, AstraZeneca, 431 83 Gothenburg, Sweden

*Corresponding author: johanna.hjalte@food.lth.se

1 *Visual appearance*

Example photographs, which illustrate the turbidity observed in some samples of ozarelix or cetrorelix, are shown in Fig. S1.

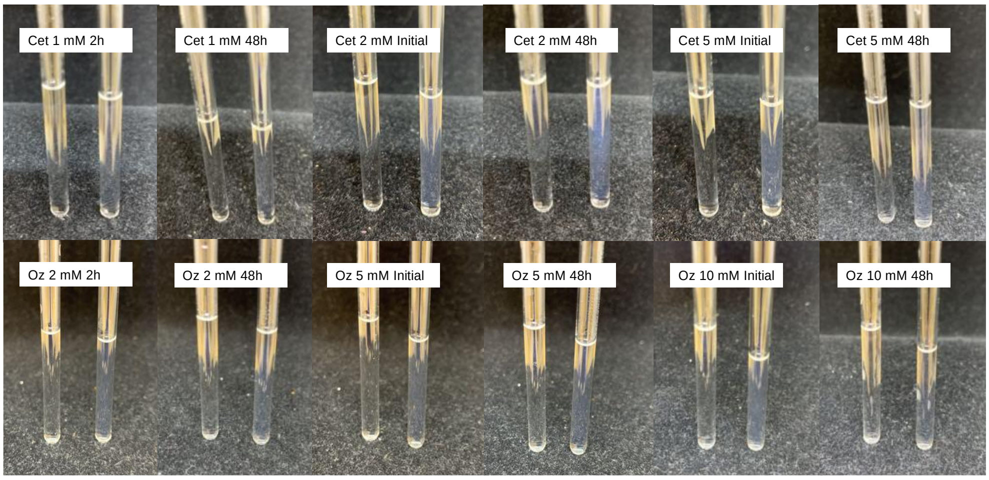

Figure S1. Photographs of selected samples. To the left of each sample is a pure water sample. All phots show some degree of light scattering. The top row shows cetrorelix samples, and the bottom row ozarelix samples. To the far left is the lowest concentration that shows scattering, at the earliest time point. For cetrorelix, the selected samples are: 1 mM at 2 h, 1 mM at 48 h, 2 mM initial, 2 mM 48 h, 5 mM initial, 5 mM 48 h. For ozarelix, the selected samples are: 2 mM at 2 h, 2 mM at 48 h, 5 mM initial, 5 mM 48 h, 10 mM initial, 10 mM 48 h.

2 *pH and charge*

The samples of D-Phe^6-GnRH, ozarelix, and cetrorelix all showed varying degrees of increase in pH, according to the resolution of pH strips (i.e. 0.5 pH units), with peptide concentration as well as over time. All samples of 0.1 mM peptide showed a pH of ~5.5, which remained stable over at least 48 h. The 10 mM samples of D-Phe^6-GnRH showed a stable pH of ~6.5 whereas samples of ozarelix and cetrorelix at the higher concentrations showed pH 7-7.5, which increased over 48 h by up to ~1 pH unit. Samples of intermediate concentrations generally showed intermediate pH values. Degarelix samples, on the other hand, had pH of ~5.5 at all concentrations and time points investigated.

At the measured pH values, peptide molecules residing in solution are, based on the pK_a values presented in Table S1 and the plots of net charge vs. pH in Fig. S2, expected to carry an (average) positive charge of +1 or somewhat higher. The actual pK_a values of the peptides, and thereby the net charges at sample pH values, can be influenced by self-assembly and variations in the solution conditions (e.g., differences in the ionic strength).

Table S1. pK_a values of the ionizable amino acid sidechains in the investigated peptides.

**Residue**	**Present in peptide types**	**Residue number**	**pK_a**	**Acid / Base**
Histidine	D-Phe^6-GnRH	2	6.0[a]	Base
3-(3-pyridinyl)-D-alanine	ozarelix, cetrorelix, degarelix	3	4.3[b]	Base
Tyrosine	D-Phe^6-GnRH, ozarelix, cetrorelix	5	10.1[a]	Acid
4-*S*-dihydroorotamido-L-phenylalanine	degarelix	5	10.2[b]	Acid
Arginine	D-Phe^6-GnRH, ozarelix, cetrorelix	8	12.1[a]	Base
N6-isopropyl-L-lysine	degarelix	8	10.8[b]	Base

a) Values for individual amino acids taken from D.R. Lide, *Handbook of Chemistry and Physics*.[1]

b) Experimental values for degarelix obtained by Yasuda-Shedlovsky extrapolation of effective pK_a values from potentiometric titration in different water-ethanol mixtures (Ferring Pharmaceuticals, internal data).

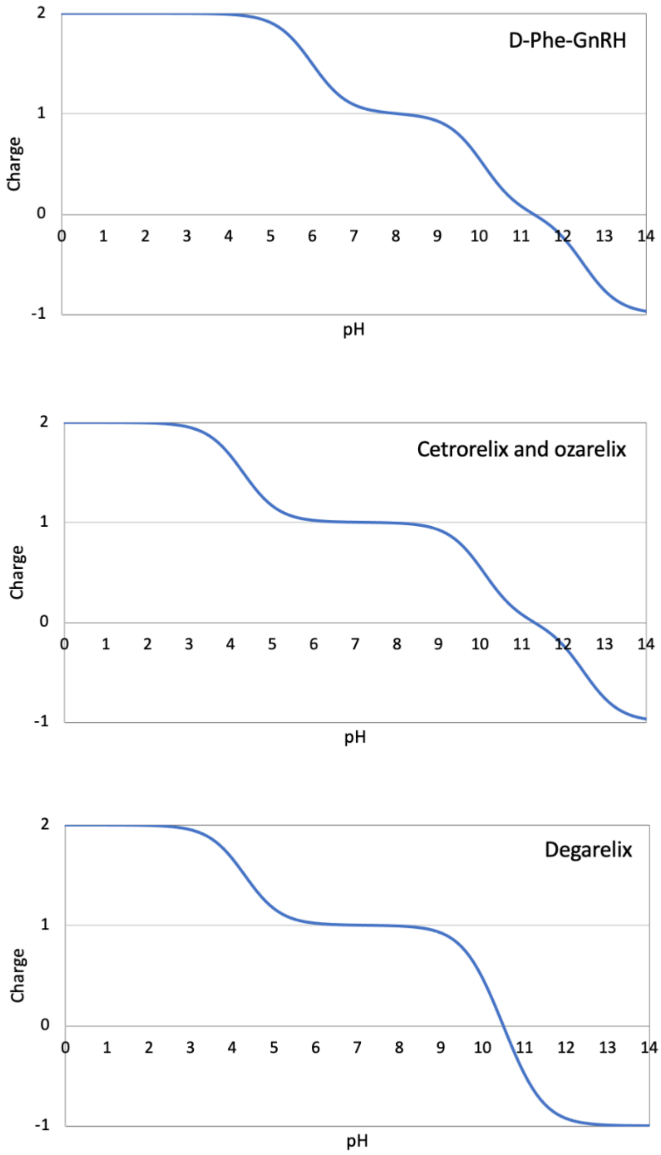

Figure S2. Plots of charge vs. pH for the investigated peptides, based on the pK_a values presented in Table S1

3 ^{1}H NMR data and comments

3.1 Comments on peak broadening in NMR spectra

Peak broadening and loss of signal can be attributed to formation of larger aggregates. The linewidth of an NMR signal depends on the spin-spin relaxation time, T_2, which, in turn, depends on the rotational correlation time, effectively the tumbling rate, of the molecule or molecular segment giving rise to an NMR signal. Generally, slower tumbling gives shorter T_2, which in turn corresponds to broader signals.[2] However, depending on the character of the self-assembled structure formed, the T_2 of a signal may be controlled largely by local mobility rather than by the tumbling rate of the aggregate as a whole. Thus, there is no general, simple relationship between aggregate size and the extent of line broadening.

The effective disappearance of signal, i.e., the reduction in absolute integral relative to the nominal concentration, that is observed with increasing concentration can be attributed to very short T_2. This can cause reduction in signal intensity by broadening so significant that the signal practically merges with the baseline, by actual loss of signal during delays in the NMR pulse sequence, or a combination. The excitation sculpting pulse sequence applied in this work for solvent suppression includes significant delay times, which will effectively function as a T_2 relaxation filter. Signal from peptide residing in larger aggregates, with short T_2, will be attenuated to a larger extent than signal from peptide present as monomers or residing in small oligomers or aggregates, which has longer T_2. Thus, features associated with monomers or small aggregates will have a higher weight in the observed NMR spectra.

3.2 ^{1}H NMR spectra

Near-complete spectra for all peptides are shown in Fig. S3. The range 4.5-6.5 ppm was excluded to allow better magnification of the more crowded aromatic region (6.5-8.5 ppm). The excluded region contained the suppressed water signal, two singlets for ozarelix and cetrorelix (at 5.4 and 6.0 ppm), and one singlet for degarelix (at 5.8 ppm). Degarelix and D-Phe^6-GnRH also showed a singlet at 9.9 and 10.0 ppm, respectively. All these peaks followed the same general development as the peaks shown in Fig. S3. There was a variability in the efficiency of water signal suppression, especially in samples with extensive aggregation and low peptide signal, resulting in relatively high intensities at 4-4.5 ppm in the spectra of 5 mM ozarelix and 10 mM cetrorelix. The excitation sculpting program might influence the intensity of signals nearby the suppressed water signal, which is one of the reasons why the leucine (or nor-leucine) signal at 0.7 ppm was selected for comparison of integral values. All spectra in Fig. S3 were individually scaled to similar intensity to show changes in line shape.

In addition to the substantial changes with concentration that are discussed in the main text a few minor variations in spectral appearance were observed among the spectra in the range above 6.5 ppm, most clearly for D-Phe^6-GnRH and degarelix at 8.2-8.5 ppm. This range show signals arising from amide protons and aromatic side chains, which can be very sensitive to variations in the local chemical environment, and these differences could be attributed to variations in the general solution conditions among samples (ionic strength, pH) just as well as to differences in direct inter-peptide interactions.

Samples of ozarelix showed an unexpected sharp singlet at 3.6 ppm at all concentrations. The origin of this signal is not fully understood but is believed to be caused by a trace contamination of 1,4 dioxan introduced in the freeze-drying process during isolation of the peptide.

D-Phe⁶-GnRH

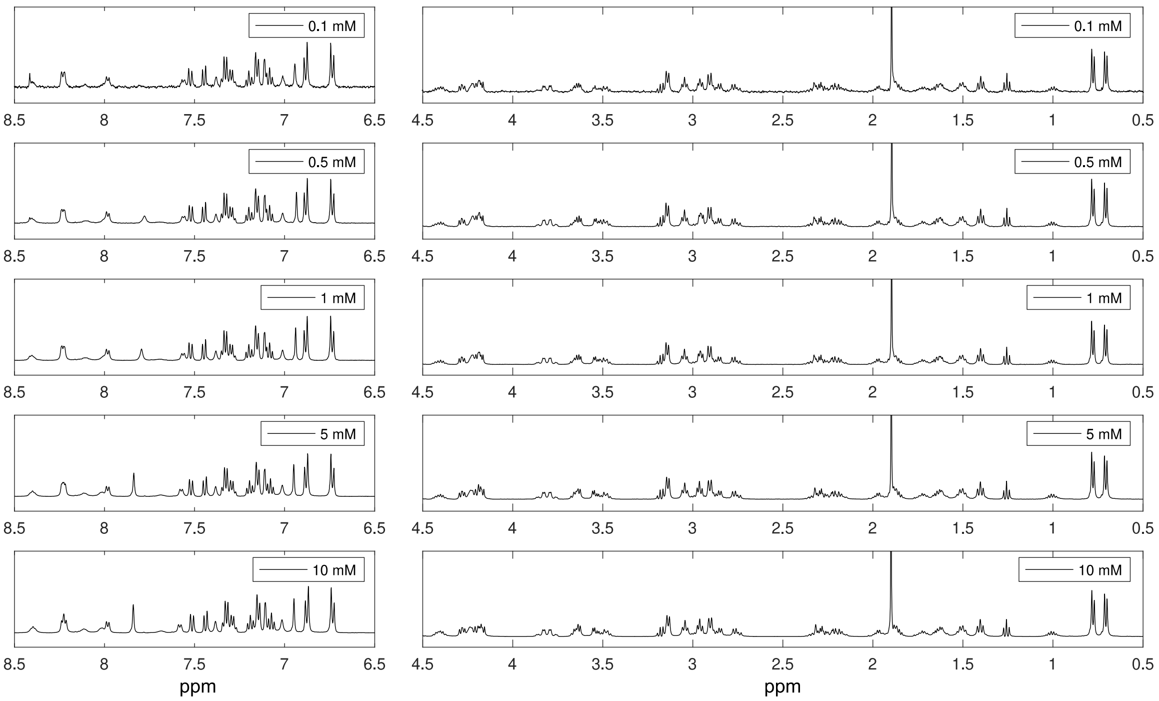

Ozarelix

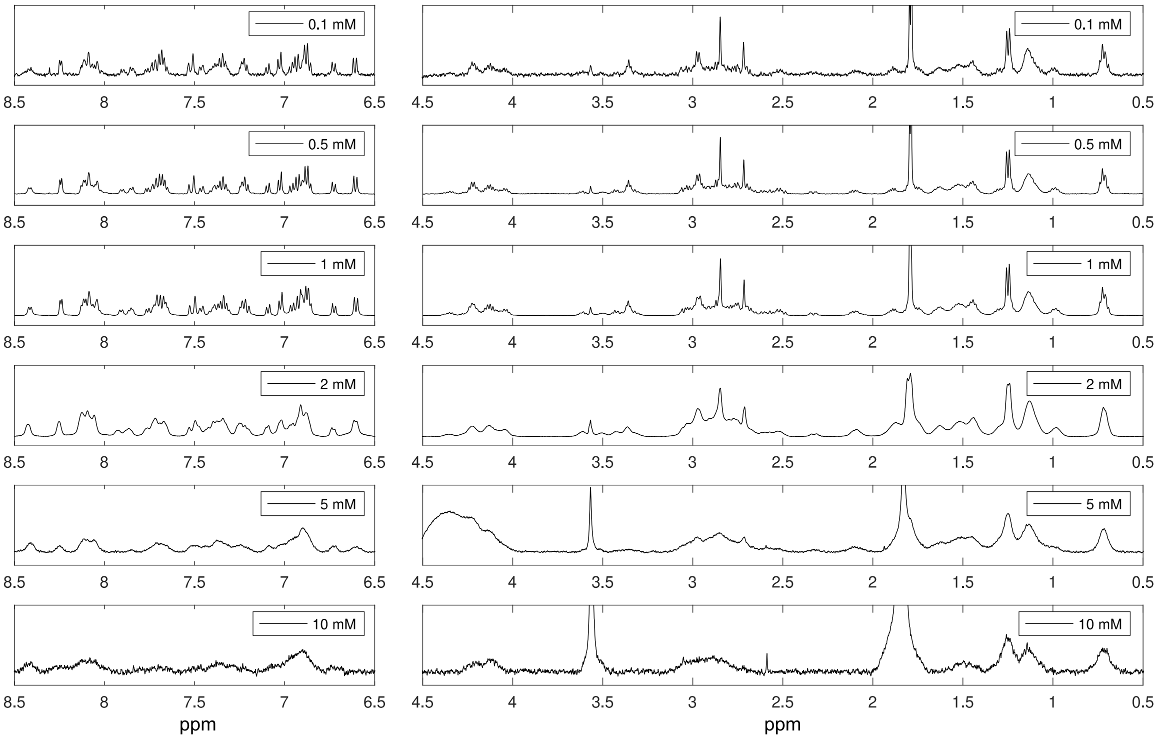

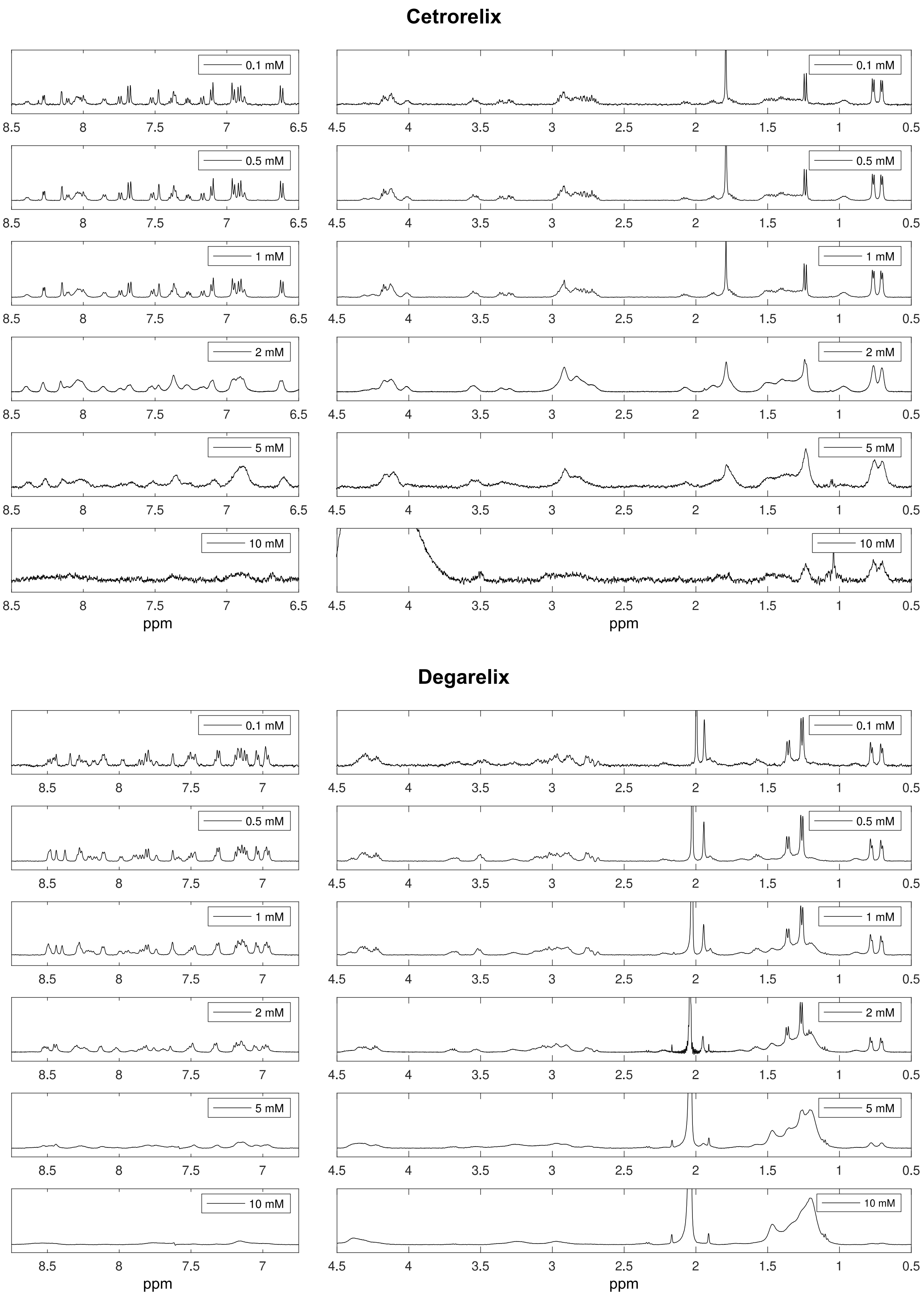

Figure S3. ^{1}H NMR spectra of D-Phe^{6}-GnRH (0.1, 0.5, 1, 5, 10 mM), and ozarelix, cetrorelix and degarelix (0.1, 0.5, 1, 2, 5, 10 mM). Each spectrum is individually scaled to show variation in spectral appearance.

3.3 Comments on the acetyl and acetate peaks

The acetyl peak, which is expected for ozarelix, cetrorelix and degarelix (but not D-Phe^{6}-GnRH) is most clearly seen in Fig. 3 (main text). The acetyl peak is expected to be close to the acetate counterion peak. For cetrorelix, there is a complete overlap (the two singlets are visible in DMSO-d6) at all concentrations where these peaks are distinguishable, whereas for ozarelix, the acetate counterion peak seems to move from the right to the left side of the acetyl peak, indicating a change in pH. Furthermore, the acetate peak seems to increase in intensity from 5 to 10 mM ozarelix, but when accounting for the concentration increase, the signals at 5 and 10 mM are most likely the result of a similar fraction of acetate in solution, while the peptide peaks disappear to a larger extent at 10 mM.

3.4 Changes in ^{1}H NMR spectra over time

For D-Phe^{6}-GnRH, the spectra remained unchanged over the investigated time interval of one week. For ozarelix, cetrorelix, and degarelix, on the other hand, several notable changes over time were observed, the most significant of which are illustrated in Fig. S4. In addition, samples of 5 and 10 mM cetrorelix showed a distinct spectrum-wide sharpening of the peaks over the investigated timespan (Fig. S4). This sharpening could be attributed to disintegration of initial large aggregates into smaller ones and/or release of free monomers. Degarelix showed a similar behavior at 5 and 10 mM, where the intensity of the group of broad signals between 1.2 and 1.6 ppm increased notably in intensity over time, whereas the remainder of the spectrum remained practically unchanged (Fig. S4).

Rapid (4 scan) experiments were performed at about 15 and 25 minutes after sample preparation, as well as after 24 hours and after a week, for all peptides at 1-10 mM concentration (spectra not included). In these experiments, the 5- and 10 mM samples of ozarelix showed substantial loss in signal intensity, without notable changes in the general spectral appearance. For 5 mM, the spectrum recorded 10 minutes after the initial spectra was about halfway to the intensity seen at the next measurement at 24 h (Fig. S4), whereas for the 10 mM sample, the spectra after 10 min and 24 h showed similar intensities These were the only samples for which a rapid, substantial reduction in signal intensity shortly after preparation was captured. The reduction in signal can likely be attributed to an initial larger fraction of relatively small aggregates, which rapidly grow into larger structures.

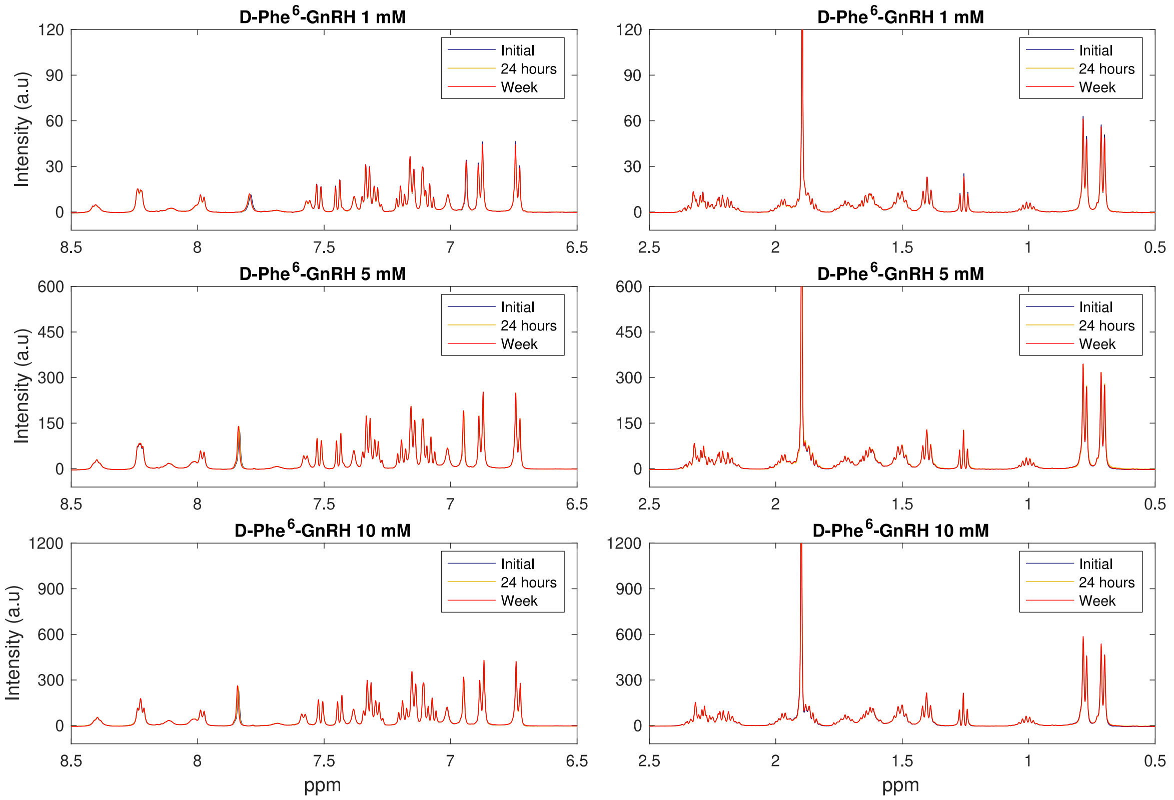
D-Phe^{6}-GnRH 1 mM
D-Phe^{6}-GnRH 5 mM
D-Phe^{6}-GnRH 10 mM
Intensity (a.u)
ppm
Initial
24 hours
Week

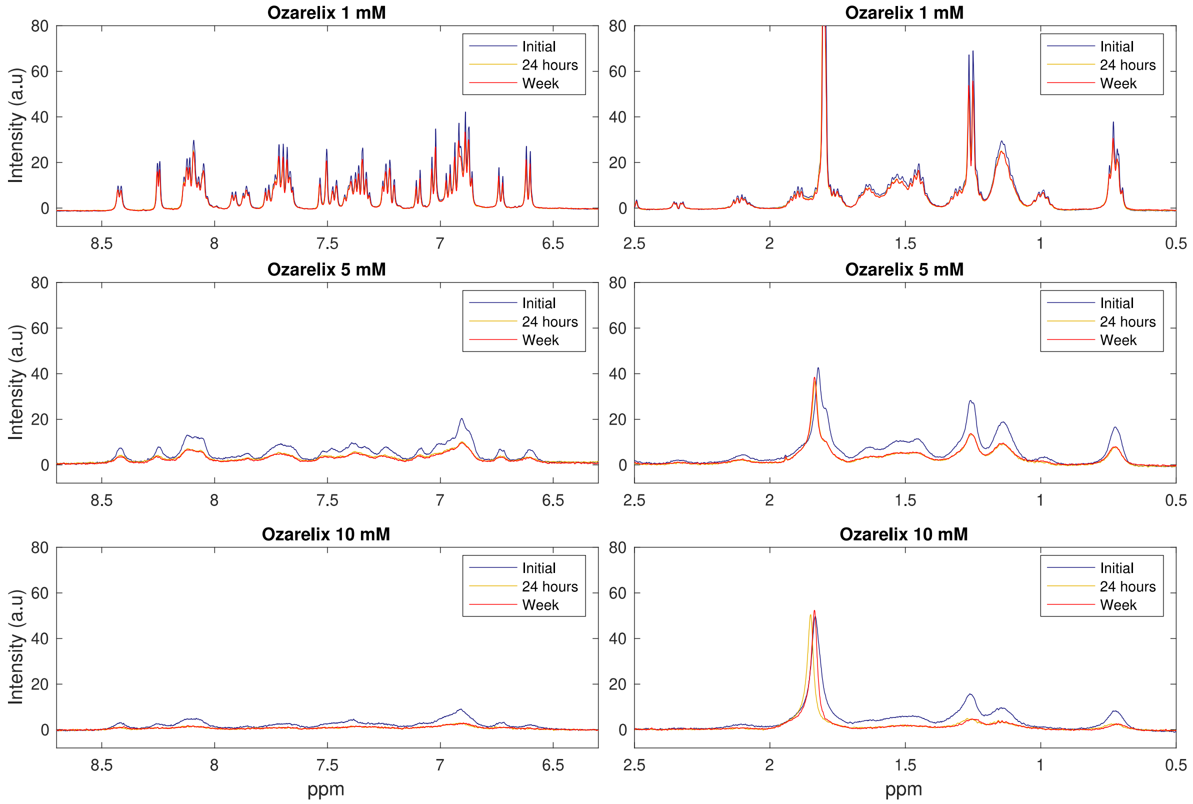
Ozarelix 1 mM
Ozarelix 5 mM
Ozarelix 10 mM
Intensity (a.u)
ppm
Initial
24 hours
Week

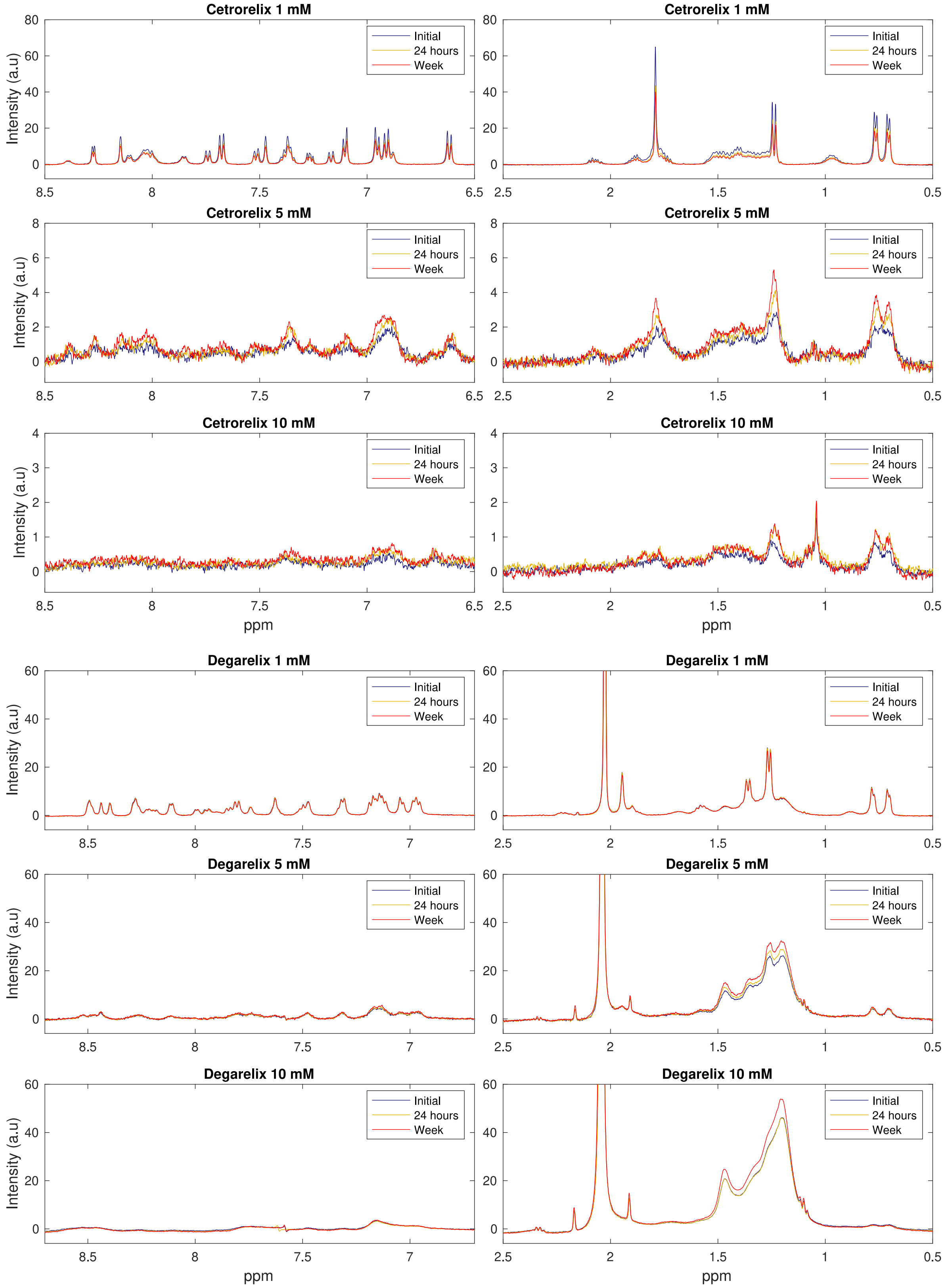

Figure S4. ^{1}H NMR spectra of D-Phe[6]-GnRH, ozarelix, cetrorelix and degarelix at 1, 5 and 10 mM, at three different times after sample preparation: initial (blue), 24 hours (yellow) and after a week (red). The same arbitrary intensity scale is used in all spectra. In all spectra line broadening is set to 1.2.

4 *NMR diffusion measurements*

4.1 Experimental details

The NMR diffusion experiments were performed at 25ºC on a Bruker AVII-200 spectrometer equipped with a Bruker DIFF-25 gradient probe and a Bruker GREAT 1/40 gradient amplifier. The temperature control was calibrated using a thermocouple immersed in an NMR tube to measure the actual temperature at the position of the sample. Samples were inserted into the probe at least 15 minutes prior to the experiments.

Self-diffusion coefficients were determined using the pulsed gradient stimulated echo (PFG-STE) method.[3] The PFG-STE sequence is preferable (compared to the simpler spin-echo experiment) in investigations of large molecules or molecules residing in large aggregates, which results in a short T_2. For a solute with an isotropic self-diffusion coefficient D, the intensity of the stimulated echo can be described by:

$$I = I_0 \exp\left[-\gamma^2 G^2 \delta^2 \left(\Delta - \frac{\delta}{3}\right) D\right] = I_0 \exp(-kD) \qquad \text{(eq. S1)}$$

where I is the intensity of the stimulated echo, I_0 is the intensity of the stimulated echo in the absence of pulsed field gradients, γ the gyromagnetic ratio of protons, G and δ the strength and duration of the gradient pulses, and Δ the diffusion time.

Experiments were run with the parameters described in Table S2 with a linear ramping of G. G was calibrated in a measurement on a trace amount of HDO in D_2O, which has a D of 1.902×10^{-9} m^2/s at 25°C.[4] For each experiment, the receiver gain was optimized using the *rga* (receiver gain adjustment) function.

Table S2. Parameters for NMR diffusion measurements.

	**D-Phe[6]-GnRH 3 mM**	**D-Phe[6]-GnRH 10 mM**	**Degarelix 3 mM**	**Degarelix 10 mM**
**Duration of gradient pulse (*d*) [ms]**	1	1	1	1
**Diffusion time (Δ) [ms]**	50	50	50	50
**Delay between first two RF pulses (τ_1) [ms]**	2.1168	2.1168	2.1568	2.2468
**Max gradient strength (*G*) [G/cm]**	179.96	179.96	359.91	569.11
**Start gradient strength (*G*) [G/cm]**	9	9	11.25	17.78
**Number of scans**	64	16	64	16
**Number of gradient steps**	16	16	32	32
**Recycle delay D1 (s)**	3.95	3.95	3.95	3.95
**Acquisition time (s)**	0.6873	0.6873	0.6873	0.6873

Self-diffusion coefficients, *D*, were estimated from the observed echo decays of relevant signals in plots of ln*I* vs. *k*, which yields *D* according to:

$$\ln I = -kD + const \qquad \text{(eq. S2)}$$

4.2 NMR diffusion theory

Under the condition of fast molecular exchange between the respective sites where a certain component can reside (with residence times much shorter than the diffusion time, Δ, of the experiments, i.e., 50 ms in this work), the observed diffusion coefficient, D_{obs}, for this component is the weighted average of the diffusion coefficients at the different sites, according to:

$$D = \alpha_1 D_1 + \alpha_2 D_2 + \alpha_3 D_3 + \ldots \qquad \text{(eq. S3)}$$

where $\alpha_{1,2,3...}$ are the fractions of the component residing at sites 1, 2, 3… and $D_{1,2,3}$... represents the corresponding diffusion coefficients (see comment about the possible influence of short values of T_2 below).

Under the condition of slow exchange (with residence times much longer than the diffusion time), on the other hand, distinct diffusion coefficients for the respective sites can be obtained. The relative contributions to the observed diffusion coefficient or coefficients may be weighted

not only by the fractions of a molecule at the respective possible sites but can also be influenced by differences in T_2, where short values of T_2 decreases the weight (caused by loss of signal during τ_1). This factor can be of particular importance in situations where there is a substantial difference in size (and thus in T_2) of different types of aggregates and/or if very large aggregates are present in the system. Very large aggregates (with very short T_2) may not be detected at all.

For a single D, which will be the outcome either if only monomers are present or if there is fast exchange between different sites (where D_{obs} is described by equation S3), the echo decay described by equations S1 and S2 will be single exponential. For multiple distinct D_i, which may result in a case of multiple sites and slow exchange, the echo decay will be multi-exponential.

The D of a molecule or an aggregate in solution can be related to the hydrodynamic radius, R_H, via the Stokes-Einstein equation:

$$D = \frac{k_B T}{6\pi\eta R_H} \qquad \text{(eq. S4)}$$

where k_B is the Boltzmann constant, T is the absolute temperature, and η the dynamic viscosity of the bulk medium.

4.3 NMR diffusion results

Samples of 3 and 10 mM of the four peptides in D_2O were analysed. Figs. S5 and S6 show examples of recorded echo decays for the 10 mM samples. All samples showed a trace signal of HDO at 4.8 ppm. Table S3 summarizes the collected NMR diffusion results.

Fig. S5 shows that D-Phe^6-GnRH at 10 mM gave a single-exponential decay, corresponding to a single D. Similar results were observed at 3 mM (not shown).

Due to the presence of large aggregates, which results in short T_2, none of the ozarelix and cetrorelix samples showed peptide signal appropriate for evaluation of diffusion coefficients. Of these samples, only the 3 mM ozarelix sample showed any peptide signal, although very

weak (not shown). Both ozarelix samples showed a very weak acetate signal, at 2 ppm (see Fig. S6a for the 10 mM sample), which was absent for the cetrorelix samples.

Degarelix at 10 mM (Fig. S5) showed signal only for the group of signals observed at 1.2-1.6 ppm and for the acetate counterion. These signals showed a multiexponential decay, indicating coexisting aggregate types with different diffusion coefficients and slow exchange. Similar results were observed at 3 mM (not shown).

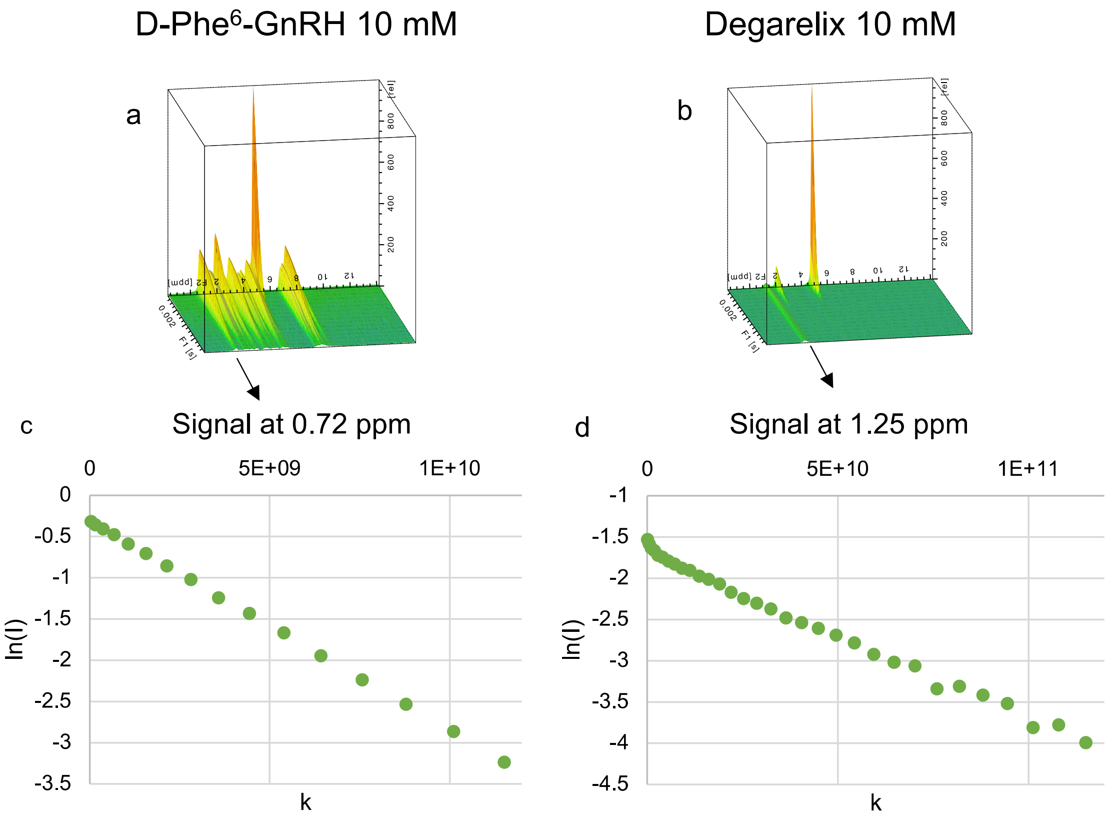

Figure S5. Development of signal intensity with increasing gradient strength during NMR diffusion measurements. Figs. S5a-b show the full studied ppm range. The signal at 4.8 ppm is from a trace amount of HDO. Figs. S6c-d show the echo decays at single ppm values, which are used to estimate the self-diffusion coefficients.

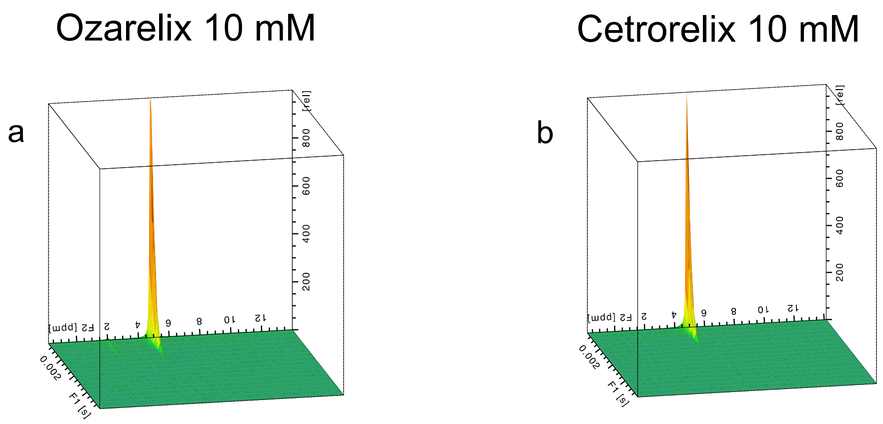

Figure S6. Development of signal intensity with increasing gradient strength over the entire studied ppm range during NMR diffusion measurements. The signal at 4.8 ppm is from a trace amount of HDO.

Table S3. Collected NMR diffusion results.

**Peptide**	**Concentration (mM)**	**Peak Position (ppm)**	**Self-diffusion Coefficient, *D*/10^{-10} (m^2/s)**
**D-Phe^6-GnRH**	3	0.75	2.0
	10	0.72	2.2
**Ozarelix**	3	N/A	N/A
	10	N/A	N/A
**Cetrorelix**	3	N/A	N/A
	10	N/A	N/A
**Degarelix**	3	1.26	0.22 – 1.6[a]
	10	1.25	0.17 – 0.56[a]

a) Multiexponential echo decay

4.4 Comments on the NMR diffusion results for D-Phe^6-GnRH

The D_{obs} obtained for the samples of 3 or 10 mM D-Phe^6-GnRH were very similar, with values corresponding to hydrodynamic radii (R_H), calculated via Eq. S4, of 0.95 and 0.89 nm (or hydrodynamic diameters of 1.9 and 1.8 nm), respectively. Comparison of these values to the extended length of the peptide molecule (3.5-4 nm, as estimated with contour lengths per amino acids of 3.5-4.0 Å) shows that the estimated effective sizes of the average diffusing entities are reasonably consistent with a single, hydrated peptide molecule.

As was mentioned in the main text, the presence of a minor fraction of small transient oligomers cannot be excluded based on the NMR diffusometry results. When differences in *D* for

monomers and aggregates are small, which is likely for loosely structured oligomers, a substantial fraction of the peptide needs to reside in the oligomers for the latter to be detected.

4.5 Comments on the NMR diffusion results for degarelix

In the diffusometry experiments on the degarelix samples, signal from the peptide itself was only detected in the range between 1.2 and 1.6 ppm, which is consistent with signals in this range being associated with a higher rate of reorientation (longer T_2). The fact that the echo decay of the signals was multiexponential means that it results from aggregates of different sizes with slow exchange. A rough evaluation of the complex echo decay suggest that it corresponds to aggregates with R_H in the range between 1 and 10 nm, which means that these may range from single molecules and/or small oligomer to aggregates containing at least tens of molecules.

5 *Additional information AA-MD simulations*

5.1 Simulation parameters and system setup

For all peptides, simulations of a single peptide placed in a cubic box with side length of 6 nm to check the single peptide conformation were performed first. Then, to investigate peptide aggregation, 20 peptides were placed in a cubic box with side length of 15 nm representing a concentration of 10 mM, keeping the peptide concentration in the simulations within the same range as the NMR studies. Three independent simulations were performed for each case with the peptides initially placed randomly in the simulation box. In all simulations, water was added to the simulation box using the Gromacs gmx solvate utility. The systems were then energy minimized using steepest descent algorithm for 10000 steps, followed by equilibration of density and pressure for 100 ps. Final production runs of 100 ns and 500 ns were then performed for single peptide and peptide assemblies, respectively. All simulations were performed using Gromacs 2016.4 with the verlet cut-off scheme.[5] Isotropic pressure coupling with a reference pressure of 1 bar (1 bar = 100 kPa) was maintained with the Parrinello-Rahman coupling method.[6] The time step was 2 fs, and system temperature was maintained at 37C by the velocity rescale thermostat.[7] Electrostatic interactions were calculated using the Particle Mesh Ewald method,[8] with a 1.2 nm short space cut-off. Van der Waals interactions were evaluated with a force-switch between 1.0 and 1.2 nm. Periodic boundary condition was also applied for all the simulations.

5.2 Temperature coupling

In all MD-simulations there are tendencies for the system energy to change by a systematic drift over time. This is caused by force truncation and integration errors, with these issues being more pronounced in single compared to double precision floating point arithmetic as well as larger time steps. To alleviate this, temperature can be kept constant by applying an algorithm that couples atoms in the system to an external heat bath of a given temperature which scales atomic velocities up or down dependent on system state.

5.3 Pressure coupling

Pressure is maintained in molecular dynamics simulations in a similar way to temperature, by coupling the system to a pressure bath, with atomic coordinates and box dimensions scaled to adjust the pressure of the system. This can be done in a few different ways, either by scaling box dimensions evenly in all directions (isotropic pressure scaling) or by allowing box vectors to vary individually (either semi-isotropic or anisotropic pressure coupling). Simulations involving lipid bilayers often use semi-isotropic pressure coupling, to differentiate pressures in the plane of the lipid bilayer (x-y) from that normal (z) to the bilayer.

6 Simulation analysis

6.1 Transition networks

The transition network was calculated using all pairwise transitions between the aggregate states using the approach described in,[9] with the exception that the different aggregate states were defined based on only the number of peptides in an assembly. After identifying all the aggregate states and the number of transitions between the states, the corresponding transition matrix was developed. For each peptide, data from all three simulations were used to build the matrix. The transition network plots were then created using the software Cytoscape3.6.[10]

6.2 Binding and unbinding

Peptide unbinding and binding events were calculated by tracking the state of each peptide - aggregated or free - at each time step (employing again the distance cut-off of 0.5 nm to separate aggregated vs free peptides). Unbinding events were defined as a change in peptide state between aggregated to free in two consecutive time steps. Similarly, for a binding event

the peptide state was changed from free to aggregated. Peptide-peptide interactions events were then characterized and quantified further by calculating the collision acceptance probability, *CAP*, using the following equation:[11, 12]

$$CAP = (n_{bind} - n_{unbind})/n_{bind} \quad \text{(eq. S5)}$$

where n_{bind} is the number of bound peptides and n_{unbind} is the number of unbound peptides in the simulation.

6.3 Contact analysis

Hydrogen bond analysis was performed using the gmx hbond utility in Gromacs. Snapshot images were produced using VMD.[13] To plot the peptide residue-residue contact map, two residues were considered to be in contact when the distance between any pair of atoms from the respective residues was under 0.5 nm.[12] Hydrophobic solvent accessible surface areas (hSASA) were computed using the Gromacs gmx sasa module. Atoms with a partial charge in the range from -0.2 to 0.2 were defined as hydrophobic and all other atoms were defined as hydrophilic.[14]

7 *Simulation results*

7.1 Collision acceptance probability (CAP)

Table S4. *CAP* values for different peptides.

**Peptide**	***CAP***
**Cetrorelix**	0.54±0.09
**Ozarelix**	0.52±0.08
**D-Phe^6-GnRH (uncharged histidine)**	0.16±0.04
**D-Phe^6-GnRH (charged histidine)**	0.03±0.01

7.2 Contact analysis results

In Figs. S7 and S8, contact analysis is used to give insight into molecular interactions within the peptide aggregates as well as between peptide and water.

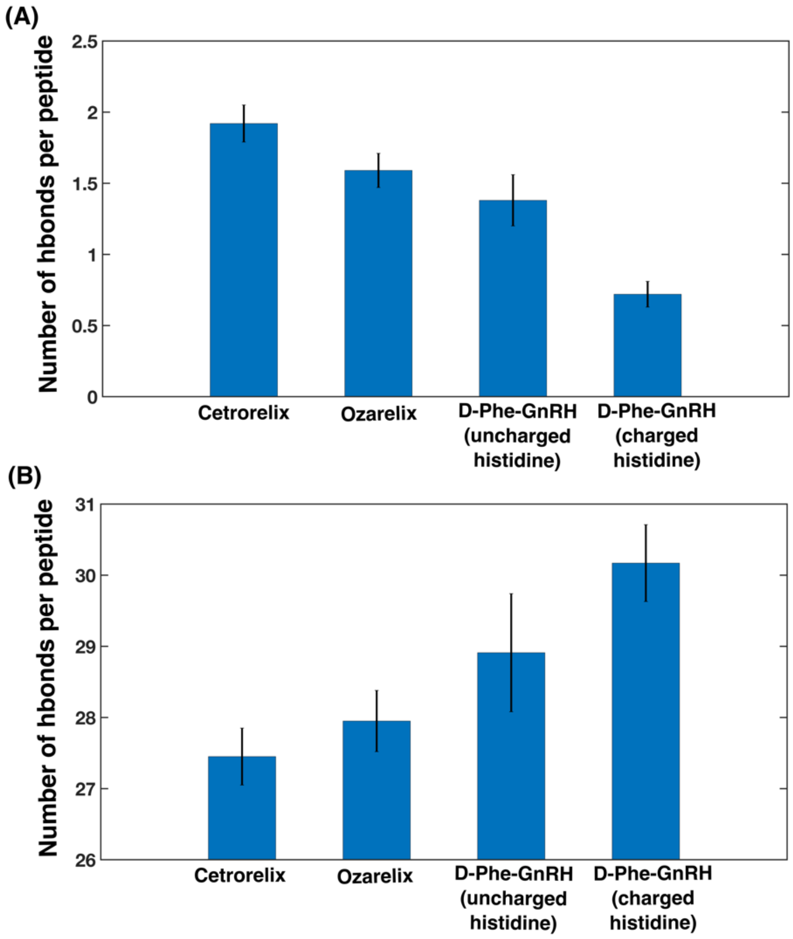

Figure S7. Number of (A) peptide-peptide and (B) peptide-water hbonds per peptide molecule averaged over the last 50 ns of each simulation.

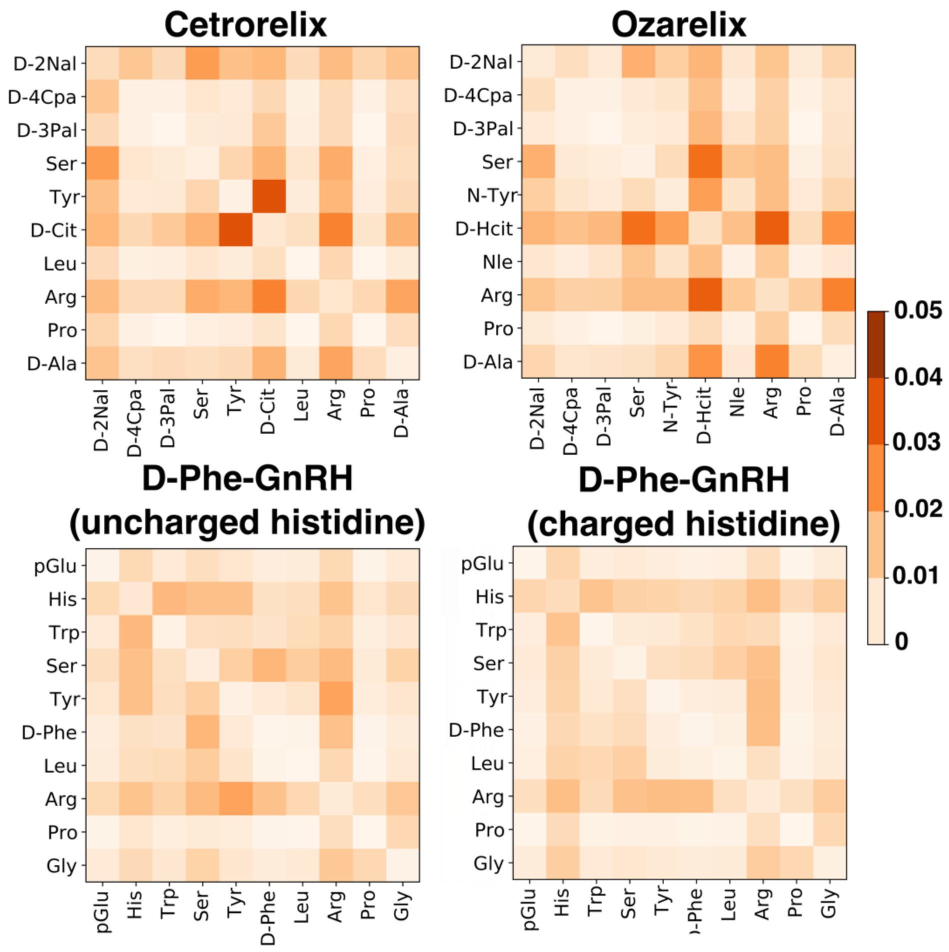

Figure S8. Inter-peptide residue-residue hbonds map. Similar to the contact map, the values are normalized by the total aggregation state of cetrorelix.

The exposure of the peptide amino acids to the solvent was characterized by assessing the hydrophobic solvent accessible surface area hSASA (Table S4).

Table S5. hSASA (Å^2) of all four different peptides for different aggregate sizes.

**Peptide**	**Aggregate Size**		
	1	2	8
**Cetrorelix**	1135 ± 41 (1135)	1973 ± 178 (986)	5905 ± 228 (738)
**Ozarelix**	1118 ± 83 (1118)	1971 ± 15 (985)	5169 ± 90 (646)
**D-Phe^6-GnRH (uncharged histidine)**	907 ±50 (907)	1667 ± 68 (833)	4377 ± 226 (547)
**D-Phe^6-GnRH (charged histidine)**	921 ± 92 (921)	1768 ± 1075 (884)	4736 ± 158 (592)

8 References

1. Lide, D. R., Biochemistry. In *CRC Handbook of Chemistry and Physics, Internet Version* 85 ed.; CRC Press: Boca Raton, FL, 2005; pp 7-1.
2. Keeler, J., Relaxation and the NOE. In *Understanding NMR spectroscopy*, 2 ed.; John Wiley & Sons Ltd: Chichester, West Sussex, United Kingdom, 2010; pp 293-301.
3. Stilbs, P. Fourier transform pulsed-gradient spin-echo studies of molecular diffusion. *Progress in nuclear magnetic resonance spectroscopy* 1987, *19*, (1), 1-45.
4. Mills, R. Self-diffusion in normal and heavy water in the range 1-45. deg. *The Journal of Physical Chemistry* 1973, *77*, (5), 685-688.
5. Abraham, M. J.; Murtola, T.; Schulz, R.; Páll, S.; Smith, J. C.; Hess, B.; Lindah, E. Gromacs: High performance molecular simulations through multi-level parallelism from laptops to supercomputers. *SoftwareX* 2015, *1-2*, 19-25.
6. Parrinello, M.; Rahman, A. Polymorphic transitions in single crystals: A new molecular dynamics method. *Journal of Applied Physics* 1981, *52*, (12), 7182-7190.
7. Bussi, G.; Donadio, D.; Parrinello, M. Canonical sampling through velocity rescaling. *Journal of Chemical Physics* 2007, *126*, (1).
8. Darden, T.; York, D.; Pedersen, L. Particle mesh Ewald: An N·log(N) method for Ewald sums in large systems. *The Journal of Chemical Physics* 1993, *98*, (12), 10089-10092.
9. Barz, B.; Liao, Q.; Strodel, B. Pathways of Amyloid-β Aggregation Depend on Oligomer Shape. *Journal of the American Chemical Society* 2018, *140*, (1), 319-327.
10. Shannon, P.; Markiel, A.; Ozier, O.; Baliga, N. S.; Wang, J. T.; Ramage, D.; Amin, N.; Schwikowski, B.; Ideker, T. Cytoscape: a software environment for integrated models of biomolecular interaction networks. *Genome research* 2003, *13*, (11), 2498-2504.
11. Carballo-Pacheco, M.; Ismail, A. E.; Strodel, B. On the Applicability of Force Fields to Study the Aggregation of Amyloidogenic Peptides Using Molecular Dynamics Simulations. *Journal of Chemical Theory and Computation* 2018, *14*, (11), 6063-6075.
12. Szała, B.; Molski, A. Aggregation kinetics of short peptides: All-atom and coarse-grained molecular dynamics study. *Biophysical Chemistry* 2019, *253*, (June), 106219-106219.
13. Humphrey, W.; Dalke, A.; Schulten, K. VMD: Visual Molecular Dynamics. *Journal of molecular graphics* 1996, *14*, (October 1995), 33-38.
14. Heller, G. T.; Aprile, F. A.; Michaels, T. C. T.; Limbocker, R.; Perni, M.; Ruggeri, F. S.; Mannini, B.; Löhr, T.; Bonomi, M.; Camilloni, C.; De Simone, A.; Felli, I. C.; Pierattelli, R.; Knowles, T. P. J.; Dobson, C. M.; Vendruscolo, M. Small-molecule sequestration of amyloid-β as a drug discovery strategy for Alzheimer's disease. *Science Advances* 2020, *6*, (45), eabb5924-eabb5924.
